# Network comparison and the within-ensemble graph distance

**DOI:** 10.1098/rspa.2019.0744

**Published:** 2020-11-04

**Authors:** Harrison Hartle, Brennan Klein, Stefan McCabe, Alexander Daniels, Guillaume St-Onge, Charles Murphy, Laurent Hébert-Dufresne

**Affiliations:** 1 Network Science Institute, Northeastern University, Boston, MA 02115, USA; 2 Laboratory for the Modeling of Biological and Socio-Technical Systems, Northeastern University, Boston, MA 02115, USA; 3 Vermont Complex Systems Center, University of Vermont, Burlington, VT 05405, USA; 4 Département de Physique, de Dénie Physique et d’Optique, Québec, Canada G1V 0A6; 5 Centre Interdisciplinaire de Modélisation Mathématique, Université Laval, Québec, Canada G1V 0A6; 6 Department of Computer Science, University of Vermont, Burlington, VT 05405, USA

**Keywords:** network comparison, graph distance, graph ensembles

## Abstract

Quantifying the differences between networks is a challenging and ever-present problem in network science. In recent years, a multitude of diverse, *ad hoc* solutions to this problem have been introduced. Here, we propose that simple and well-understood ensembles of random networks—such as Erdős–Rényi graphs, random geometric graphs, Watts–Strogatz graphs, the configuration model and preferential attachment networks—are natural benchmarks for network comparison methods. Moreover, we show that the expected distance between two networks independently sampled from a generative model is a useful property that encapsulates many key features of that model. To illustrate our results, we calculate this *within-ensemble graph distance* and related quantities for classic network models (and several parameterizations thereof) using 20 distance measures commonly used to compare graphs. The within-ensemble graph distance provides a new framework for developers of graph distances to better understand their creations and for practitioners to better choose an appropriate tool for their particular task.

## 1. Introduction

Quantifying the extent to which two finite graphs structurally differ from one another is a common, important problem in the study of networks. We see attempts to quantify the dissimilarity of graphs in both theoretical and applied contexts, ranging from the comparison of social networks [1–3], to time-evolving networks [4–8], biological networks [5], power grids and infrastructure networks [9], object recognition [10], video indexing [11] and much more. Together, these network comparison studies all seek to define a notion of dissimilarity or *distance* between two networks and to then use such a measure to gain insights about the networks in question.

However, it is often unclear which network features a given graph distance will or will not capture. For this reason, rigorous benchmarks must be established in order to better understand the tendencies and biases of these distances. We adopt the perspective that random graph ensembles are the appropriate tool to achieve this task. Specifically, by sampling pairs of graphs from within a given random ensemble with the same parameterization and measuring the graph distance between them, we create a benchmark that allows us to better understand the sensitivity of a given graph distance to known statistical features of an ensemble. Ultimately, a good benchmark would characterize the behaviour of graph distances between graphs sampled from both *within* an ensemble and *between* different ensembles. We tackle the former in this paper, noting a rich diversity of behaviours among commonly used graph distance measures. Even though this work focuses on within-ensemble graph distances, these results guide our understanding of how any two sets of networks structurally differ from each other regardless of whether those sets are generated by the same random ensemble or another network-generating process. Put simply, the approach introduced in this work is general and can be used to develop a number of graph distance benchmarks.

There are many approaches used to quantify the dissimilarity between two graphs, and we highlight 20 different ones here. Given the large number of algorithms considered in this work, we find it useful to systematically characterize each of these measures. We do so by breaking them down into ‘description-distance’ pairs. That is, every graph distance measure can be thought of as (i) computing some *description* or property of two graphs and (ii) quantifying the difference between those descriptions using some *distance* metric.

### (a) Formalism of graph distances

#### (i) Graph descriptors

**Definition 1.1.**

A *graph description* Ψ is a mapping from a set of graphs G to a space D,

1.1 Ψ:G→D.

The set G is that of all finite labelled simple graphs, and the space D is known as the *graph descriptor space*. Typically, D is $R^{l\times m}$ for integers *l*, *m* or is a space of probability distributions. Given a description Ψ, the *descriptor* of graph *G*, denoted *ψ*_*G*_, is the element of D to which *G* is mapped; *ψ*_*G*_ = Ψ(*G*).

#### (ii) Descriptor distances

**Definition 1.2.**

A distance maps a pair of descriptors to a non-negative real value,

1.2 $$d:D\times D\to R_{+}$$

and satisfies the following properties for all x,y∈D:

(i) *d*(*x*, *y*) = *d*(*y*, *x*) (symmetry)
(ii) *d*(*x*, *x*) = 0 (identity law)

The properties listed in this definition are general and mirror those in the literature [12]. They do not restrict the large possibility of measures we might use, while also providing a clean separation between how we choose to describe graphs and how we calculate the differences between those descriptions. A common property when considering distance measures is the *triangle inequality*; however, we have not included this in the list above as not all commonly used graph distances obey this property [13]. As in the case of pseudometrics, *d*(*x*, *y*) = 0 does not always imply *x* = *y* [7].^1^

#### (iii) Graph distances

**Definition 1.3.**

Given a set of graphs M⊆G, a graph description Ψ, its descriptor space D, and a distance *d* on D, the associated graph distance measure $D:M\times M\to R_{+}$ is a function defined by

1.3 $$D(G,G^{\prime })=d(\psi_{G},\psi_{G^{\prime }}).$$

Every graph distance quantifies some notion of dissimilarity between two graphs.^2^

#### (iv) Network spaces

**Definition 1.4.**

Given a distance *d* and description Ψ on descriptor space D and a set of graphs M⊆G, the associated network space, denoted (d,Ψ,M), is the set of descriptors mapped to by Ψ from graphs in M, equipped with *d* as a distance measure.

The network space (d,Ψ,M) consists of |M| points in D, namely ${\left\{\psi_{G}\right\}}_{G\in M}\subseteq D$—giving rise to |M|(|M|+1)/2 distance values, one for each pair of descriptions of elements of M.

Fundamental questions naturally arise. Does a network space capture known properties of a given ensemble of graphs? This question we can begin to answer by considering sets of graphs with known properties: i.e. random graph models.

#### (v) Models

**Definition 1.5.**

A model $M_{\vec{\alpha }}$ is a process which generates a probability distribution $P_{\vec{\alpha }}$ over a set of graphs M⊆G, where $\vec{\alpha }$ is a vector of parameters needed by the model to generate the distribution.

Models (or null models) represent a collection of maximally random graphs constrained by a set of parameters, which we use to generate sets of graphs [14]. The probability distribution of model $M_{\vec{\alpha }}$ is then defined over the set of graphs that have non-zero probability of being generated given the model and its parameters $\vec{\alpha }$. For many well-known models, we have a deep understanding of how the structure of sampled graphs is influenced by the parameter values. Using our knowledge of how parameters affect graph structure, we can see how well the expected features of a given model are reflected by the structure of each network space.

### (b) This study

Herein, we apply a variety of graph distances to pairs of independently and identically sampled networks from a variety of random network models, over a range of parameter values for each, and consider the within-ensemble distance distribution as a function of the type of graph and model parameters. While our focus is on the means of the distance distributions, we also include the standard deviations in each figure. Ultimately, we report the within-ensemble graph distances for 20 different graph distances from the software package, netrd.^3^ To our knowledge, this is the largest systematic comparison of graph distances to date.

## 2. Methods

### (a) Ensembles

We study the behaviour of (d,Ψ,M) for sets of graphs sampled from $M_{\vec{\alpha }}$ under a variety of parameterizations. There are many graph ensembles that one could use to compute within-ensemble graph distances, and we begin by focusing on two broad classes: ensembles that produce graphs with homogeneous degree distributions and those that produce graphs with heterogeneous degree distributions. In total, we study the within-ensemble graph distance for five different ensembles.

#### (i) ErdőLgs–Rényi random graphs

Graphs sampled from the ErdőLgs–Rényi model (ER), also known as *G*_(*n*,*p*)_, have (undirected) edges among *n* nodes, with each pair being connected with probability *p* [15,16]. This model is commonly used as a benchmark or a null model to compare with observed properties of real-world network data from nature and society. In our case, it allows us to explore the behaviour of graph distance measures on dense and homogeneous graphs. In fact, this model maximizes entropy subject to a global constraint on expected edge density, *p*.

One well-studied construction of this ensemble is when *p* = 〈*k*〉/*n*, in which *n* nodes are connected uniformly at random such that nodes in the resulting graph have an average degree of 〈*k*〉. This ensemble is particularly useful for identifying which graph distance measures are able to capture key structural transitions that happen as the average degree increases. For convenience, we will refer to this ensemble as *G*_(*n*,〈*k*〉)_.

#### (ii) Random geometric graphs

We work with random geometric graphs of *n* nodes and edge density *p*, generated by sprinkling *n* coordinates uniformly into a one-dimensional ring of circumference 1, and connecting all pairs of nodes whose coordinate distance (arc length) is less than or equal to *p*/2. Compared to *G*_(*n*,*p*)_, this model produces graphs that have a high average local clustering coefficient, which is a property commonly found in real network data. Note that setting the connection distance to *p*/2 means that *p* parameterizes the edge density exactly as in *G*_(*n*,*p*)_ [17,18].

#### (iii) Watts–Strogatz graphs

Watts–Strogatz (WS) graphs allow us to study the effects that random, long-range connections have on regular lattices. A WS graph is initialized as a one-dimensional regular ring-lattice, parameterized by the number of nodes *n* and the even-integer degree of every node 〈*k*〉 (each node connects to the 〈*k*〉/2 closest other nodes on either side). Each edge in the network is then randomly rewired with probability *p*_*r*_, which generates graphs with both relatively high average clustering and relatively short average path lengths for a wide range of *p*_*r*_ ∈ (0, 1) [19].

#### (iv) (Soft) configuration model with power-law degree distribution

We generate *expected* degree sequences from distributions with power-law tails with a mean of 〈*k*〉. We construct an instance of a ‘soft’ configuration model, the maximum entropy network ensemble with a given sequence of expected degrees, by connecting node-pairs with probabilities determined via the method of Lagrange multipliers [20–22]. Through this method, we are able to construct networks with a tunable degree exponent, *γ*. The degree exponents that we test range from those that skew the distribution heavily, resulting in a highly heterogeneous ultra-small-world network (*γ* ∈ (2, 3)), to those that generate more homogeneous networks (*γ* > 3). In contrast to the homogeneous ensembles we tested—all of which have homogeneous degree distributions—the requirement of heterogeneity in these graphs constrains the possible edge densities to be vanishingly small. Otherwise, in the high-edge density regime, degrees cannot fluctuate to appreciably larger-than-average values, and we have a natural degree scale imposed by the network size.

#### (v) Nonlinear preferential attachment

The final ensemble of networks included here are grown under a degree-based nonlinear preferential attachment mechanism [23–25]. A network of *n* nodes is grown as follows: each new node is added to the network sequentially, connecting its *m* edges to nodes already in the network *v*_*i*_ ∈ *V* with probability $\Pi_{i}=k_{i}^{\alpha }/\sum_{j}k_{j}^{\alpha }$, where *k*_*i*_ is the degree of node *v*_*i*_ and *α* modulates the probability that a given node already in the network will collect new edges. When *α* = 1, this model generates networks with a power-law degree distribution (with degree exponent *γ* = 3), and a condensation regime emerges as *n* → ∞ when *α* > 2, producing a star network with *O*(*n*) nodes all connected to a main hub node [25].

### (b) Graph distance measures

The study of network similarity and graph distance has yielded many approaches for comparing two graphs [5,26]. Typically, these methods involve comparing simple descriptors based on either aggregate statistical properties of two graphs—such as their degree or average path length distributions [4]—or intrinsic spectral properties of the two graphs, such as the eigenvalues of their adjacency matrices, or of other matrix representations [27]. The description distances also tend to fall into two broad categories: either classic definitions of norms or distances based on statistical divergence. While different approaches are better suited for capturing differences between certain types of graphs, they obviously are expected to share several properties.

The simplest graph distances aggregate element-wise comparisons between the adjacency matrices of two graphs [28–31] and extensions thereof [32]; these methods depend explicitly on the node labelling scheme (and hence are not invariant under graph isomorphism [33]), which may limit their utility when comparing graphs with unknown labels (e.g. graphs sampled from random graph ensembles, as we do here). Several measures collect empirical distributions [34] or a ‘signature’ vector [1] from each graph and take the distance between them (using the Jensen–Shannon divergence, Canberra distance, earth mover’s distance etc.^4^ ), which, among other things, facilitates comparison of differently sized graphs [4,36]. Another family of approaches compare spectral properties of certain matrices characterized by the graphs [37], such as the non-backtracking matrix [7,38] or Laplacian matrix [27]. The relevant spectral properties associated with these distances are invariant under graph isomorphism [33,39]. Some graph distances have been shown to be metrics (i.e. they satisfy properties such as triangle inequality, etc.) [13], whereas others have not. These are not exhaustive descriptions of every graph distance in use today, but they represent coarse similarities between the various methods. We summarize the 20 graph distances we consider in table 1 and more extensively define them in electronic supplementary material, B.

**Table 1.** Graph distances. Distance measures used to systematically compare graphs in this work, as well as their abbreviated labels, and their sources. Lap., Laplacian; Gauss., Gaussian; Loren, Lorenzian; JSD, Jensen–Shannon divergence; Euc., Euclidian distance.

	graph distance	label
1	Jaccard [29]	JAC
2	Hamming [30]	HAM
3	Hamming–Ipsen–Mikhailov [37]	HIM
4	Frobenius [28]	FRO
5	polynomial dissimilarity [5]	POD
6	degree JSD [34]	DJS
7	portrait divergence [4]	POR
8	quantum spectral JSD [40]	QJS
9	communicability sequence [41]	CSE
10	graph diffusion distance [42]	GDD
11	resistance perturbation [8]	REP
12	NetLSD [3]	LSD
13	Lap. spectrum; Gauss. kernel, JSD [27]	LGJ
14	Lap. spectrum; Loren. kernel, Euc. [27]	LLE
15	Ipsen–Mikhailov [43]	IPM
16	non-backtracking eigenvalue [7]	NBD
17	distributional non-backtracking [38]	DNB
18	D-measure distance [9]	DMD
19	DeltaCon [2]	DCN
20	NetSimile [1]	NES

### (c) Description of experiments

See table 2 for the full parameterization of these sampled graphs. In each experiment, we generate *N* = 10^3^ pairs of graphs for every combination of parameters. With these sampled random graphs, we measure the distance between pairs from the same parameterization of the same model, $M_{\vec{\alpha }}$, and report statistical properties of the resulting vectors of distances. In other words, our experiments consist of calculating mean within-ensemble graph distances,

2.1 $$\langle D\rangle =\sum_{G,G^{\prime }\in G}D(G,G^{\prime })P_{\vec{\alpha }}(G)P_{\vec{\alpha }}(G^{\prime }),$$

where $P_{M,\vec{\alpha }}:G\to [0,1]$ (or $P_{\vec{\alpha }}$ when its meaning is unambiguous) is the graph probability distribution for model $M_{\vec{\alpha }}$. This is estimated by sampling *N* ≫ 1 graph-pairs ${\left\{(G_{i},G_{i}^{\prime })\right\}}_{i=1}^{N}$ and computing

2.2 $$\langle D\rangle \approx \frac{1}{N}\sum_{i=1}^{N}D(G_{i},G_{i}^{\prime }).$$

**Table 2.** Experiment parameterization. Here, we report the ensembles that were used in these experiments, as well as their parameterizations. For *G*_(*n*,〈*k*〉)_ and WS key parameters, we span 100 values, spaced logarithmically, between the values above. *Parameter labels*: *n*, network size; *p* = density; 〈*k*〉 = average degree; *p*_*r*_, probability that a random edge is randomly rewired; *γ*, power-*law degree exponent*; *α*, preferential attachment kernel. Note: In electronic supplementary material, A, we show how the within-ensemble graph distance changes as *n* increases.

ensemble	fixed parameter(s)	key parameter
*G*_(*n*,*p*)_	*n* = 500	*p* ∈ {0.02, 0.06, …, 0.98}
RGG	*n* = 500	*p* ∈ {0.02, 0.06, …, 0.98}
*G*_(*n*,〈*k*〉)_	*n* = 500	〈*k*〉 ∈ {10^−4^, …, *n*}
WS	*n* = 500, 〈*k*〉 = 8	*p*_*r*_ ∈ {10^−4^, …, 10^0^}
SCM	*n* = 1000, 〈*k*〉 = 12	*γ* ∈ {2.01, 2.06, …6.01}
PA	*n* = 500, 〈*k*〉 = 4	*α* ∈ { − 5, − 4.95, …, 5}

We then study the behaviour of 〈*D*〉 for various $M_{\vec{\alpha }}$. The error on the mean within-ensemble graph distance is estimated from the following standard error of the mean $\sigma_{\left\langle D\right\rangle }\approx \sigma_{D}/\sqrt{N}$, where *σ*_*D*_ is the standard deviation on the within-ensemble graph distance *D*, estimated by sampling as well. For all experiments, we used *N* = 10^3^ pairs of graphs, which is sufficient in general as can be seen from the small standard error relative to the mean in all figures. In each plot, we also include the standard deviations *σ*_*D*_ of the within-ensemble graph distances, and we highlight when the standard deviation offers particularly notable insights into the behaviour of certain distances.

Lastly, there are several distances that assume alignment in the node labels of *G* and *G*′. Because we are sampling from random graph ensembles, the networks we study here are not node-aligned, and as such, care should be taken when interpreting the output of these graph distances. For every description of graph distances in electronic supplementary material, B, we note if node alignment is assumed.

## 3. Results

In the following sections, we broadly describe the behaviour of the mean within-ensemble graph distance (in general denoted 〈*D*〉) for the distance measures tested. The general structure of this section is motivated by critical properties of the ensembles studied here. We highlight features of the within-ensemble graph distance for two broad characterizations of networks: homogeneous and heterogeneous graph ensembles, focusing on specific ensembles within each category.

All of the main results from the experiments described below are summarized in table 3, which practitioners may find especially useful when considering which tools to use for comparing networks with particular structures. When relevant, we highlight certain distance measures to emphasize interesting within-ensemble graph distance behaviours.

**Table 3.** Summary of key within-ensemble graph distance properties for different ensembles. Each ensemble included in this work has characteristic properties that a within-ensemble graph distance may be able to capture. Here, we consolidate these various properties into a single table that classifies whether each distance has a given property. Models considered are dense Erdős-Rényi graphs (*G*_(*n*,*p*)_), random geometric graphs (RGG), sparse Erdős–Rényi graphs (*G*_(*n*,〈*k*〉)_), the Watts–Strogatz model (WS), soft configuration model with power-law degree distribution (SCM) and general preferential attachment with kernel *α* (PA). *Clarifications*: In the WS model, we look at three properties: (i) the mean within-ensemble graph distance is larger for intermediate ‘small-world’ values of *p*_*r*_ than it is when *p*_*r*_ = 1; (ii) the within-ensemble graph distance is sensitive to values of *p*_*r*_ where the magnitude slope of the *L*_*p*_/*L*_0_ curve is largest (‘path length sensitivity’ above); (iii) the within-ensemble graph distance is sensitive to values of *p*_*r*_ where the magnitude slope of the *C*_*p*_/*C*_0_ curve is largest (‘clustering sensitivity’ above). In the PA model, we look at whether high, positive values of *α* produce greater mean within-ensemble graph distances than lower, negative values of *α*, and at where the maximum within-ensemble distance occurs.

model	property	JAC	HAM	HIM	FRO	POD	DJS	POR	QJS	CSE	GDD	REP	LSD	LGJ	LLE	IPM	NBD	DNB	DMD	DCN	NES
*G*_(*n*,*p*)_	complement symmetry		✓	✓	✓	✓	✓														
*G*_(*n*,*p*)_	derivative with network size, *n*	0	0	0	+	—	—	—	∼	—	∼	—	—	—	—	—	+	—	—	+	∼
RGG	maximum: $p\approx \frac{1}{2}$		✓	✓	✓	✓	✓	✓								✓					
*G*_(*n*,〈*k*〉)_	detects the giant 1-core							${✓}^{*}$		${✓}^{*}$	✓		✓	${✓}^{*}$	✓	✓	${✓}^{*}$	✓	${✓}^{*}$		
*G*_(*n*,〈*k*〉)_	detects the giant 2-core							${✓}^{*}$		${✓}^{*}$									${✓}^{*}$		${✓}^{*}$
*G*_(*n*,〈*k*〉)_	derivative with network size, *n*	0	—	—	+	—	—	∼	0	—	+	+	+	—	—	—	∼	—	—	+	—
WS	small-world > random							✓		✓		✓	✓	✓	✓	✓			✓		
WS	path length sensitivity						${✓}^{*}$	✓			✓	✓		${✓}^{*}$			✓	✓	✓	${✓}^{*}$	✓
WS	clustering sensitivity									✓	✓		✓	✓	✓	✓		${✓}^{*}$			${✓}^{*}$
SCM	maximum: 2 < *γ* < 3	✓	✓	✓	✓	✓	✓	✓	✓	✓	✓	✓	✓	✓	✓	✓	✓	✓	✓		✓
SCM	monotonic decay as *γ* grows		${✓}^{†}$	✓	${✓}^{†}$	${✓}^{†}$	✓	${✓}^{†}$	${✓}^{†}$		✓		✓	✓	✓	✓	${✓}^{†}$	✓	✓		${✓}^{†}$
PA	heterogeneous > homogeneous									✓	✓										
PA	maximum: *α* ≈ 0 (uniform)	✓	✓		✓	✓															
PA	maximum: *α* ≈ 1 (linear)								✓			✓									
PA	maximum: 1 < *α* ≤ 2			✓			✓	✓		✓			✓	✓	✓	✓	✓	✓	✓		✓

✓= captures a given property through a global maximum/minimum in its within-ensemble graph distance curve. ∼=non-monotonic relationship between network size and within-ensemble graph distance. ${✓}^{*}=$ potentially captures a given property (via local maxima in the mean or standard deviation, change in slope etc.). ${✓}^{†}=$ monotonic decay beyond a very small value of *γ* (*γ* ≈ 2) where there is an apparent maximum (for SCM).

### (a) Results for homogeneous graph ensembles

#### (i) Dense graph ensembles

Here, we present our results for the two models that produce homogeneous and dense graphs.

The *G*_(*n*,*p*)_ model possesses three notable features that we might expect graph distance measures to recover. Note that while we might expect graph distances to recover these features, we are not asserting that every graph distance measure *should* capture these properties.

(i) The size of the ensembles shrink to a single isomorphic class in the limits *p* → 0 and *p* → 1, corresponding respectively to an empty and complete graph of size *n*. In both limits, we might therefore expect 〈*D*(*M*_*n*,*p*_)〉 to go to zero for any method that considers unlabelled graphs.
(ii) The *G*_(*n*,*p*)_ model creates ensembles of graphs and graph complements symmetric under the change of variable *p*′ = 1 − *p*. By definition, every graph *G* has a complement $\bar{G}$ such that every edge that does (or does not) exist in *G* does not (or does) exist in $\bar{G}$. Therefore, for every graph in *G*_(*n*,*p*)_, one can expect to find its complement occurring with the same probability in *G*_(*n*,1−*p*)_. We might expect 〈*D*(*M*_*n*,*p*_)〉 = 〈*D*(*M*_*n*,1−*p*_)〉 if graph distances can capture this symmetry.
(iii) A density of $p=\frac{1}{2}$ produces the *G*_(*n*,*p*)_ ensemble with maximal entropy (all graph configurations have an equal probability). As a result, we might also expect 〈*D*(*M*_*n*,*p*_)〉 to have a global maximum at $p=\frac{1}{2}$.

The RGG model shares features 1 and 3 with the *G*_(*n*,*p*)_ model, but not feature 2. Moreover, the most significant differences between the two models is that edges are not independent in the RGG model. Correlations between edges lead to local structure (i.e. higher-order structures like triangles) and to correlations in the degree distribution. We therefore do not expect distance measures focused on the degree distribution to produce exactly the same mean within-ensemble distance curve in RGG as in *G*_(*n*,*p*)_. Conversely, any distance measure that does produce the exact same within-ensemble distance curve for RGG and *G*_(*n*,*p*)_ either fails to account for these correlations, or the effect of these correlations is negligible on the overall distance between two graphs drawn from the ensemble. This is the case for HAM, HIM and FRO.

**Figure 1.**
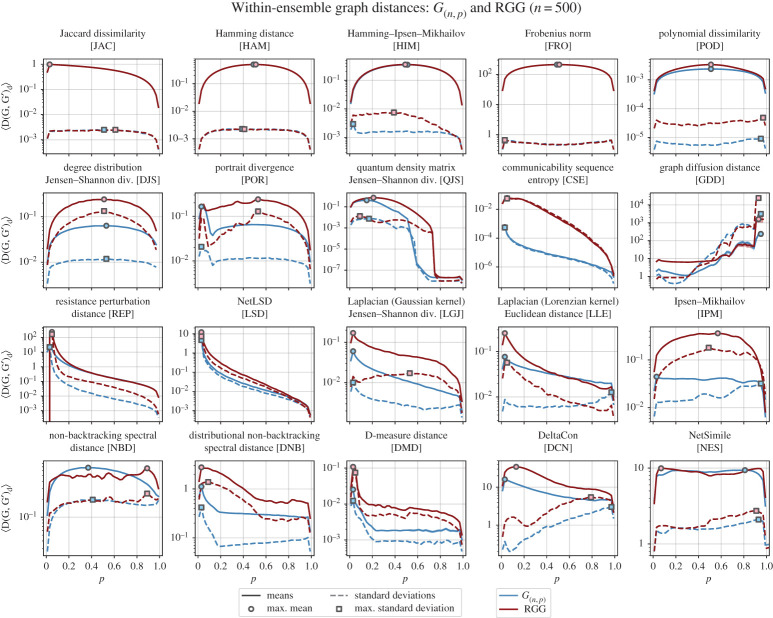
Mean and standard deviations of the within-ensemble distances for *G*_(*n*,*p*)_ and RGG. By repeatedly measuring the distance between pairs of *G*_(*n*,*p*)_ and RGG networks of the same size and density, we begin to see characteristic behaviour in both the graph ensembles as well as the graph distance measures themselves. In each subplot, the mean within-ensemble graph distance is plotted as a solid line with a shaded region around for the standard error (〈*D*〉 ± *σ*_〈*D*〉_; note that in most subplots above, the standard error is too small to see), while the dashed lines are the standard deviations. (Online version in colour.)

Our results for homogeneous graph ensembles are shown in figure 1. Only 5 out of 20 graph distances capture all the features discussed above, namely: HAM, HIM, FRO, POD, DJS. Notably, these are some of the simplest methods considered. In fact, these include two in which theoretical predictions for ER graphs precisely match the observed results for both ER graphs and RGGs, despite no consideration of RGGs having been included in such calculations. In one case (FRO), ER graphs and RGGs behave identically, yet there is also an *n*-dependence (see electronic supplementary material, figure A1).

#### (ii) Sparse graph ensembles

While the previous section highlighted dense RGG and ER networks, we now turn to the within-ensemble graph distance of *sparse* homogeneous graphs sampled from *G*_(*n*,*p*)_, such that *p* = 〈*k*〉/*n*. In the case of sparse graphs, the edge density decays to zero in the *n* → ∞ limit as the mean degree 〈*k*〉 remains fixed. We found it important to cast this distinction between dense *G*_(*n*,*p*)_ because of critical transitions that take place as 〈*k*〉 increases. As network scientists, these early transition points in sparse networks are foundational, with implications for a number of network phenomena (i.e. the occurrence of outbreaks in disease models [44], etc.).

In fact, the presence of such critical transitions in random graph models underscores the utility of this approach for studying graph distance measures. That is, a sudden change in the within-ensemble graph distance signals abrupt changes in the probability distribution over the set of graphs in the ensemble (i.e. the emergence of novel graph structures that are markedly different from the greater population of graphs in an ensemble). This may show up as a local or global maximum within-ensemble graph distance near parameter values for which this transition occurs. Conversely, if a sudden decrease in within-ensemble graph distance is observed, then there may be a sudden disappearance or reduction in largely dissimilar graphs in the ensemble.

In the case of *G*_(*n*,*p*)_ where *p* = 〈*k*〉/*n*, which we will refer to with the shorthand, *G*_(*n*,〈*k*〉)_, the following critical transitions emerge:

(iv) At 〈*k*〉 = 1, we see the emergence of a giant component in ER networks (likewise, a 2-core emerges at 〈*k*〉 = 2). We might expect, for example, a within-*G*_(*n*,〈*k*〉)_ graph distance to have a local maximum at such values.

**Figure 2.**
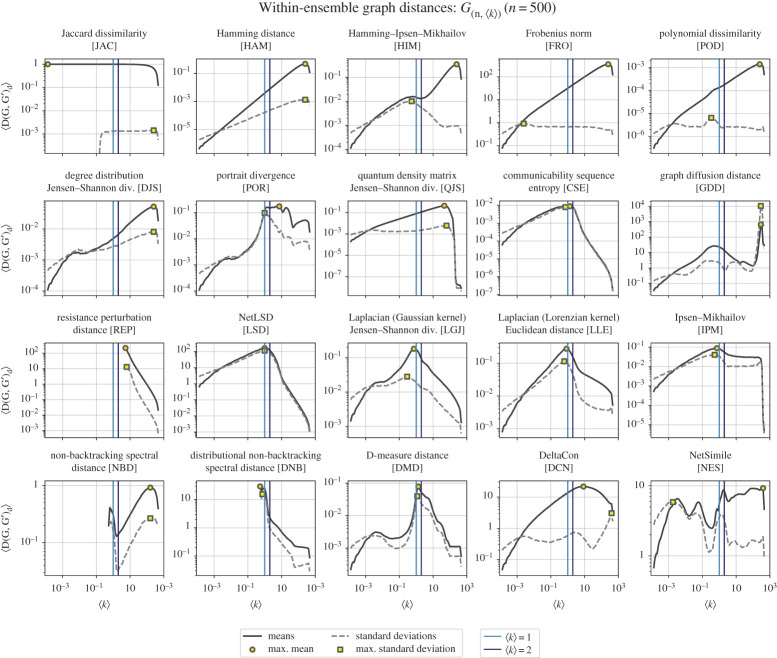
Mean and standard deviations of the within-ensemble distances for *G*_(*n*,〈*k*〉)_ networks. Here, we generate pairs of ER networks with a given average degree, 〈*k*〉, and measure the distance between them with each distance measure. In each subplot, we highlight 〈*k*〉 = 1 and 〈*k*〉 = 2. In each subplot, the mean within-ensemble graph distance is plotted as a solid line with a shaded region around for the standard error (〈*D*〉 ± *σ*_〈*D*〉_; note that in most subplots above, the standard error is too small to see), while the dashed lines are the standard deviations. (Online version in colour.)

Ultimately, we observe that distance measures that are fundamentally associated with flow-based properties of the network (i.e. if a distance measure is based on a graph’s Laplacian matrix, communicability, or other properties important to diffusion, such as path-length distributions etc.) are the ones most sensitive for picking up on this property (figure 2).^5^

What figure 2 highlights, which the dense ensembles in figure 1 could not, is the rich and varied behaviour characteristic of sparse graphs. For example, the distance measures with maxima at $p=\frac{1}{2}$ (HAM, HIM, FRO, POD, DJS, etc.) are still seen in figure 2, but the emphasis is instead on the degree as opposed to the edge density; given that most real-world networks are sparse [45], this view of the same parameter is especially informative.

Importantly, while the qualitative behaviours discussed here are general features of the models and distances, the quantitative value of the average within-ensemble graph distance also depends on network size. There are no specific structural transitions to discuss around this dependency, but it can be an important problem when comparing networks of different sizes without a good understanding of how network distances might behave. Interested readers can find our results in electronic supplementary material, A, where we use *G*_(*n*,〈*k*〉)_ to vary network size while keeping all other features fixed.

#### (iii) Small-world graphs

**Figure 3.**
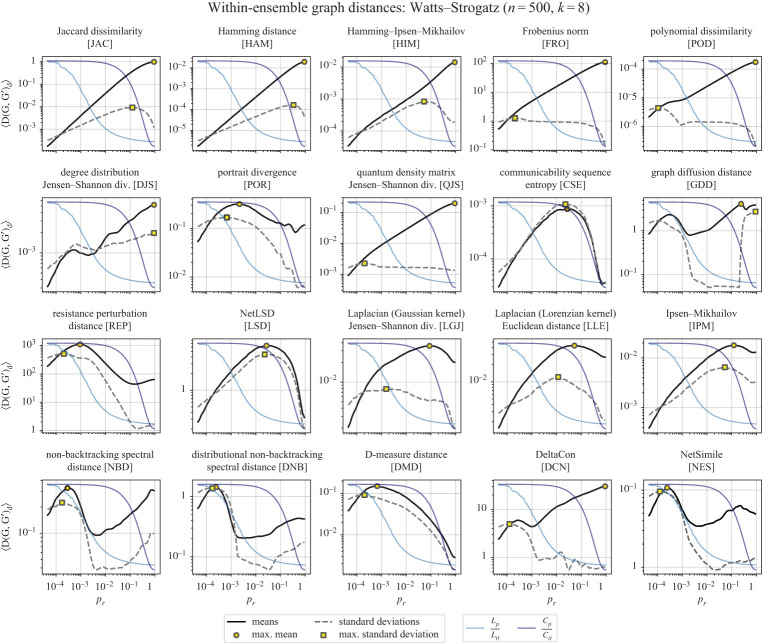
Mean and standard deviations of the within-ensemble distances for Watts–Strogatz networks. Here, we generate pairs of Watts–Strogatz networks with a fixed size and average degree but a variable probability of rewiring random edges, *p*_*r*_. In each subplot, we also plot the clustering and path length curves as in the original Watts–Strogatz paper [19] to accentuate the ‘small-world’ regime with high clustering and low path lengths. The mean within-ensemble graph distance is plotted as a solid line with a shaded region around for the standard error (〈*D*〉 ± *σ*_〈*D*〉_; note that in most subplots above, the standard error is too small to see), while the dashed lines are the standard deviations. (Online version in colour.)

The final homogeneous graph ensemble studied here is the WS model. This model generates networks that are initialized as lattice networks, and edges are randomly rewired with probability, *p*_*r*_. At certain values of *p*_*r*_, we see two key phenomena occur:

(v) ‘Entry’ into the small-world regime: Even as the edges in the network are minimally rewired, the average path length quickly decreases relative to its initial (longer) value. This is highlighted by the blue curve in figure 3, corresponding to *L*_*p*_/*L*_0_, where *L*_0_ is the average path length before any edges have been rewired. For the parameterizations used in this study, the largest (negative) slope of this curve is at *p*_*r*_ ≈ 2 × 10^−3^. We might expect a within-ensemble graph distance to be sensitive to this or nearby values of *p*_*r*_, as this region corresponds to changes in the graphs’ common structural features.
(vi) ‘Exit’ from the small-world regime: After enough edges have been rewired, the network loses whatever clustering it had from originally being a lattice, reducing to approximately the clustering of an ER graph. This is highlighted by the violet curve in figure 3, corresponding to *C*_*p*_/*C*_0_, where *C*_0_ is the average clustering before any edges have been rewired. For the parameterizations used in this study, the largest (negative) slope of this curve is at *p*_*r*_ ≈ 3 × 10^−1^. Again, we might expect a within-ensemble graph distance to be sensitive to this large decrease in clustering.

Together, the above features characterize WS networks. Importantly, we are interested in whether a distance measure is *sensitive* to these ‘entry’ and ‘exit’ values of *p*_*r*_; sensitive here is deliberately broadly defined. For instance, as in the case of CSE, we observe a reduction in within-ensemble graph distance at a rate that almost exactly resembles the rate at which *C*_*p*_/*C*_0_ decays. Alternatively, a distance measure can be sensitive to these critical points by having a local maximum at or around the critical point. In the case of POR, we see that the within-ensemble graph distance is maximized at approximately the same point as the largest (negative) slope of the *L*_*p*_/*L*_0_ curve.

Here, *insensitivity* to these critical points is also an informative property to highlight in a distance measure. As one example, HAM appears to be otherwise unaffected by the ‘exit’ from the small-world regime, with distances increasing steadily despite the model generating networks with dramatic structural differences.

**Figure 4.**
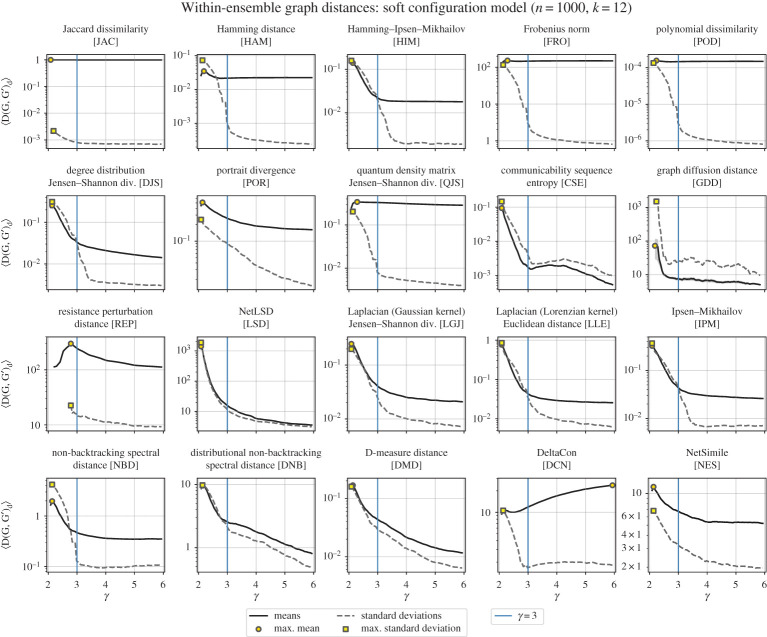
Mean and standard deviations of the within-ensemble distances for soft configuration model networks with varying degree exponent. Here, we generate pairs of networks from a (soft) configuration model, varying the degree exponent, *γ*, while keeping 〈*k*〉 constant (*n* = 1000). In each subplot, we highlight *γ* = 3. The mean within-ensemble graph distance is plotted as a solid line with a shaded region around for the standard error (〈*D*〉 ± *σ*_〈*D*〉_; note that in most subplots above, the standard error is too small to see), while the dashed lines are the standard deviations.

Lastly, we ask whether the within-ensemble graph distance of random networks (i.e. when *p*_*r*_ → 1) is greater than that of small-world networks; this is indicated by a within-ensemble graph distance curve that is higher at *p*_*r*_ = 1 than those between 10^−3^ < *p*_*r*_ < 10^−1^ in figure 3. This property holds for distance measures that depend on node labelling (e.g. JAC, HAM, HIM, FRO, POD) but also for DJS—which is intuitive, since more noise increases the variance of the degree distribution—as well as a few puzzling distances: QJS, DCN and the two based on the non-backtracking matrix, NBD and DNB (figure 4).

### (b) Results for sparse heterogeneous ensembles

The sparse graph setting is much closer to that of real networks, which often also have heavy-tailed degree distributions [46]. This motivated the selection of the following two heterogeneous, sparse ensembles.

#### (i) Soft configuration model: heavy-tailed degree distribution

We study these graphs using a (soft) configuration model with a power-law expected degree distribution; i.e. the expected degree *κ* of a node is drawn proportionally to *κ*^−*γ*^. From this model, we expect two important features that graph distance measures could recover:

(vii) For *γ* < 3, we know the variance of the degree diverges in the limit of large graph size *n* [46]. Since there should be large variations on the degree sequences for two finite instances, we might also expect the graph distances to produce maximal distance 〈*D*〉.
(viii) We might also expect a *monotonic* decay in the within-ensemble graph distance as *γ* increases. For large *γ*, most expected node-degrees will be approximately the average degree, making the network as a whole structurally similar to an ER graph. On the other hand, when *γ* is small (especially when *γ* ≤ 3), there is a wide diversity in the degrees of nodes within the graph, and of the expected degrees of nodes across graphs (since expected degrees are i.i.d. sampled from a Pareto distribution).

Out of the 20 studied, most distances capture both of these features. Since *γ* tunes the degree-heterogeneity (larger *γ* yielding more homogeneous graphs), a decrease in the average distance among pairs of graphs might be expected. For large *γ*, most expected node-degrees will be approximately the average degree, making the network as a whole structurally similar to an ER graph. On the other hand, when *γ* is small (especially when *γ* ≤ 3), there is a wide diversity in the degrees of nodes within the graph, and of the expected degrees of nodes across graphs (since expected degrees are i.i.d. sampled from a Pareto distribution). Thus a reasonable expectation would be that pairs of graphs on average become further apart as *γ* is decreased. This is observed in many distances, but with the exceptions of QJS and REP, which each instead exhibit maxima at certain finite values of *γ* > 2. Additionally, several distances (HAM, POR, NBD and NES) appear to decay monotonically beyond some very small value of *γ*, below which they have a slightly smaller value. This fact could have arisen as a finite-size effect or due to some other details of the implementation, since fluctuations become highly pronounced as *γ* → 2.

Only one graph distance produces completely unexpected behaviour: DCN yields 〈*D*〉 that monotonically increases with the scale exponent *γ* of the degree distribution, and its standard deviation is *minimized* when *γ* ≈ 3. We will expand upon this in the following section.

#### (ii) Nonlinear preferential attachment

The final ensemble we include here is the nonlinear preferential attachment growth model. By varying the preferential attachment kernel, parameterized by *α*, we can capture a range of network properties:

(ix) As *α* → −∞, this model generates networks with maximized average path lengths, whereby each new node connects its *m* links to nodes with the smallest average degree; conversely *α* → ∞ generates star-like networks [47], an effect known as *condensation*.
(x) At *α* = 1, linear preferential attachment, we see the emergence of scale-free networks [23], whereas uniform attachment *α* = 0 gives each node an equal chance of receiving the incoming node’s links.

**Figure 5.**
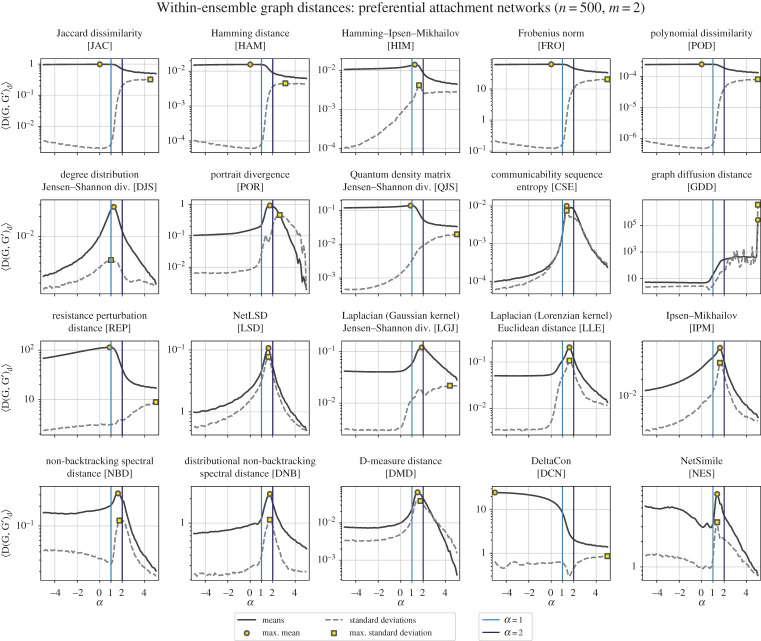
Mean and standard deviations of the within-ensemble distances for preferential attachment networks. Here, we generate pairs of preferential attachment networks, varying the preferential attachment kernel, *α*, while keeping the size and average degree constant. As *α* → ∞, the networks become more and more star-like, and at *α* = 1, this model generates networks with power-law degree distributions. The mean within-ensemble graph distance is plotted as a solid line with a shaded region around for the standard error (〈*D*〉 ± *σ*_〈*D*〉_; note that in most subplots above, the standard error is too small to see), while the dashed lines are the standard deviations. (Online version in colour.)

When *α* = 1, this ensemble theoretically generates networks with power-law degree distributions (with degree exponent, *γ* = 3 [24]), which is reminiscent of the results in figure 5 where we measure the within-ensemble graph distances while varying *γ*.

Various mean within-ensemble distances are maximized in the range *α* ∈ [1, 2], which is indicative of the diversity of possible graphs that can be produced by the preferential attachment mechanism in the small-*α* regime. For *α* ≪ 0, newly arriving nodes connect primarily to the lowest-degree existing nodes (for example, leading to long chains of degree-2 nodes when *m* = 1), making many distance measures record i.i.d. pairs of graphs as similar. For *α* ≫ 0, new nodes tend to connect to the highest-degree existing node, leaving a star-like network—then likewise many graph-pairs are deemed very similar. In the intermediate range (e.g. linear preferential attachment, *α* = 1), a much wider variety of possible graphs can arise. Thus on average, i.i.d. pairs are (usually) measured as furthest apart in that range.

For preferential attachment networks, we again see curious behaviour for DCN where, unlike most other distance measures, heterogeneous graphs with 1 ≤ *α* < 2 have smaller within-ensemble graph distances than more homogeneous graphs *α* < 0. Upon closer examination, we know why this happens, and to conclude this section, we will walk through the anatomy of DCN and show why its behaviour is often different than the other distance measures studied here, especially for heterogeneous networks.

The *descriptor*, *ψ*_*G*_ that DCN is based off of is an *affinity matrix* of the graph (constructed from a belief propagation algorithm, see electronic supplementary material, B, for full methodology), while the *distance* is calculated using the Matusita distance (similar to the Euclidean distance). The authors note that they selected this distance because they found that it gave more desirable results: ‘·· ·it “boosts” the node affinities and, therefore, detects even small changes in the graphs (other distance measures, including [Euclidean distance], suffer from high similarity scores no matter how much the graphs differ)’ [2]. What the choice of the Matusita distance has apparently obscured, however, is a greater specificity for distinguishing heterogeneous networks. We know this because of preliminary experiments where the Matusita distance is swapped out for a Jensen–Shannon divergence (as in, for example, CSE); this resulting within-ensemble graph distance *is* maximized for heterogeneous networks (1 < *α* < 2).

Finally, as we note in §i, we are not asserting that a graph distance measure *should* detect the unique behaviour of linear preferential attachment (*α* = 1). Nor are we advocating for practitioners to abandon the use of DCN. What we are claiming, however—and why we chose to focus on DCN in this section—is that we need useful benchmarks for understanding the effects of choosing one descriptor-distance pairing over another. Furthermore, this benchmark *should* be based on the within-ensemble graph distances from well-known ensembles.

## 4. Discussion

Graph ensembles are core to the characterization and broader study of networks. Graphs sampled from a given ensemble will highlight certain observable features of the ensemble itself, and in this work, we have used the notion of graph distance to further characterize several commonly studied graph ensembles. The present study focused on one of the simplest quantities to construct given a distance measure and a graph ensemble, namely the mean within-ensemble distance 〈*D*〉. Note, however, that there are many ensembles for which the present methods could be repeated, as well as more graph distance measures, and infinitely many other statistics that could be examined from the within-ensemble distance distribution. Despite examining the within-ensemble graph distances for only five different ensembles, we observed a richness and variety of behaviours among the various distance measures tested. We view this work as the starting point for more inquiries into the relationship between graph ensembles and graph distances.

One promising future direction for the study of within-ensemble graph distances is the prospect of deriving functional forms for various distance measures, as we do for JAC, HAM and FRO in electronic supplementary material, C. Other distance measures, such as DJS, likely have approximate analytical expressions derived for certain graph ensembles.

We have here only studied the behaviour of graphs within a given ensemble and parameterization, which is essentially the simplest possible choice. This leaves wide open any questions regarding distances between graphs sampled from *different* ensembles—or even different from two different parameterizations of the same ensemble. These will be the topic of follow-up works. Nevertheless, such follow-ups will likewise only cover a very small fraction of all possible combinations.

We hope that our approach will provide a foundation for researchers to clarify several aspects of the network comparison problem. First, we expect that practitioners will be able to use the within-ensemble graph distance in order to *rule out* suboptimal distance measures that do not pick up on meaningful differences between networks in their domain of interest (e.g.what is an informative ‘description-distance’ comparison between brain networks may not be as informative when comparing, for example, infection trees in epidemiology). Second, we expect that this work will provide a foundation for researchers looking to develop new graph distance measures (or hybrid distance measures, such as HIM) that are more appropriate for their particular application areas.

There were 20 different graph distances used in this work, with undoubtedly more that we have not included. Each of these measures seek to address the same thing: quantifying the dissimilarity of pairs of networks. We see the current work as an attempt to consolidate all such methods into a coherent framework—namely, casting each distance measure as a mapping of two graphs into a common descriptor space, and the application of a distance measure within that space. Not only that, we also suggest that stochastic, generative, graph models—because of known structural properties and certain critical transition points in their parameter space—are the ideal tool to use for characterizing and benchmarking graph distance measures.

Classic random graph models can fill an important gap by providing well-understood benchmarks on which to test distance measures *before* using them in applications. Much like in other domains of network science, having effective and well-calibrated comparison procedures is vital, especially given the great diversity of graph ensembles under study and of networks in nature.

## Supplementary Material

Supplementary Information 1

## Footnotes

1 For example, two cospectral but non-identical graphs would have distance zero according to any spectral distance measure.

2 Throughout this paper, we use the term ‘graph distance’ or ‘distance’ to refer to a dissimilarity measure between two graphs satisfying the properties we detail in §a. This language is somewhat imprecise from a mathematical perspective; many graph distances do not meet all the criteria of distance *metrics*. We have chosen to keep the term ‘graph distance’ at the cost of some informality to maintain consistency with much of the existing literature we draw upon. We thank one of the anonymous reviewers for their help in clarifying this matter.

3 See https://github.com/netsiphd/netrd/. This software package includes several more distances that were not included in these analyses, and as it is an open-source project, we anticipate that it will be updated with new distance measures as they continue to be developed.

4 From our preliminary analyses, the particular choice of metric can dramatically change the distance values, though we do not report this here. For an extensive description of distance metrics in general, see [12,35].

5 Note that the two distance measures based on the non-backtracking matrix (NBD and DNB) are undefined in graphs without a 2-core, restricting their range in figure 2.

## Data accessibility

The experiments in this paper were conducted using the netrd Python package github.com/netsiphd/netrd [48]. Replication materials can be found at github.com/jkbren/wegd.

## References

[1] Berlingerio M, Koutra D, Eliassi-Rad T, Faloutsos C 2012 NetSimile: a scalable approach to size-independent network similarity. (http://arxiv.org/abs/1209.2684)

[2] Koutra D, Vogelstein JT, Faloutsos C 2016 DeltaCon: principled massive-graph similarity function with attribution. ACM Trans. Knowl. Discov. Data 10, 1–43. (10.1145/2824443)

[3] Tsitsulin A, Mottin D, Karras P, Bronstein A, Müller E 2018 NetLSD: Hearing the shape of a graph. In *Proc. of the 24th ACM SIGKDD Int. Conf. on Knowledge Discovery & Data Mining*. 2347–2356. (doi:10.1145/3219819.3219991).

[4] Bagrow J, Bollt E 2019 An information-theoretic, all-scales approach to comparing networks. Appl. Netw. Sci. 45, 1–15. (10.1007/s41109-019-0156-x)

[5] Donnat C, Holmes S 2018 Tracking network dynamics: a survey using graph distances. Ann. Appl. Stat. 12, 971–1012. (10.1214/18-AOAS1176)

[6] Masuda N, Holme P 2019 Detecting sequences of system states in temporal networks. Sci. Rep. 9, 795 (10.1038/s41598-018-37534-2) 30692579 PMC6349888

[7] Torres L, Suárez-Serrato P, Eliassi-Rad T 2019 Non-backtracking cycles: Length spectrum theory and graph mining applications. Appl. Netw. Sci. 4, 41 (10.1007/s41109-019-0147-y)

[8] Monnig N, Meyer F 2018 The resistance perturbation distance: a metric for the analysis of dynamic networks. Discrete Appl. Math. 236, 347–386. (10.1016/j.dam.2017.10.007)

[9] Schieber T, Carpi L, Díaz-Guilera A, Pardalos P, Masoller C, Ravetti M 2017 Quantification of network structural dissimilarities. Nat. Commun. 13928, 1–10. (10.1038/ncomms13928) PMC5227707 28067266

[10] Wilson R, Zhu P 2008 A study of graph spectra for comparing graphs and trees. Pattern Recognit. 41, 2833–2841. (10.1016/j.patcog.2008.03.011)

[11] Bunke H, Shearer K 1998 A graph distance metric based on the maximal common subgraph. Pattern Recognit. Lett. 19, 255–259. (10.1016/S0167-8655(97)00179-7)

[12] Deza MM, Deza E 2009 Encyclopedia of distances. New York, NY: Springer (esp. Section 1.1, pp. 3–10).

[13] Bento J, Ioannidis S 2019 A family of tractable graph distances. Appl. Netw. Sci. 4, 1–27. (10.1007/s41109-019-0219-z)

[14] Newman M 2018 Networks. Oxford, UK: Oxford University Press.

[15] Erdős P, Rényi A 1959 On random graphs. Publicationes Mathematicae 6, 290–297. (10.2307/1999405)

[16] Bollobás B 1980 A probabilistic proof of an asymptotic formula for the number of labelled regular graphs. Eur. J. Comb. 4, 311–316. (10.1016/S0195-6698(80)80030-8)

[17] Dall J, Christensen M 2002 Random geometric graphs. Phys. Rev. E 66, 016121 (10.1103/PhysRevE.66.016121) 12241440

[18] Penrose M 2003 Random geometric graphs. Oxford, UK: Oxford University Press.

[19] Watts D, Strogatz S 1998 Collective dynamics of ‘small-world’ networks. Nature 393, 440–442. (10.1038/30918) 9623998

[20] Park J, Newman M 2004 Statistical mechanics of networks. Phys. Rev. E 70, 066117 (10.1103/PhysRevE.70.066117) 15697444

[21] Garlaschelli D, Loffredo M 2008 Maximum likelihood: extracting unbiased information from complex networks. Phys. Rev. E 78, 015101 (10.1103/PhysRevE.78.015101) 18764006

[22] Cimini G, Squartini T, Saracco F, Garlaschelli D, Gabrielli A, Caldarelli G 2019 The statistical physics of real-world networks. Nat. Rev. Phys. 1, 58–71. (10.1038/s42254-018-0002-6)

[23] Barabási A, Albert R 1999 Emergence of scaling in random networks. Science 286, 509–512. (10.1126/science.286.5439.509) 10521342

[24] Albert R, Barabási A 2002 Statistical mechanics of complex networks. Rev. Mod. Phys. 74, 47–97. (10.1103/RevModPhys.74.47)

[25] Krapivsky PL, Redner S, Leyvraz F 2000 Connectivity of growing random networks. Phys. Rev. Lett. 85, 4629–4632. (10.1103/RevModPhys.74.47) 11082613

[26] Wills P, Meyer F 2020 Metrics for graph comparison: a practitioner’s guide. PLoS ONE 15, e0228728 (10.1371/journal.pone.0228728) 32050004 PMC7015405

[27] Jurman G, Visintainer R, Furlanello C 2011 An introduction to spectral distances in networks. *Neural Nets WIRN10: Proceedings of the 20th Italian Workshop on Neural Nets*. 227–234. (10.3233/978-1-60750-692-8-227)

[28] Golub G, van Loan C 2013 Matrix computations. Baltimore, MD: JHU Press 1421407949 9781421407944.

[29] Jaccard P 1901 Étude de la distribution florale dans une portion des Alpes et du Jura. Bull. de la Societe Vaudoise des Sciences Naturelles 37, 547–579. (10.5169/seals-266450)

[30] Hamming RW 1950 Error detecting and error correcting codes. Bell Syst. Tech. J. 29, 147–160. (10.1016/S0016-0032(23)90506-5)

[31] Gao X, Xiao B, Tao D, Li X 2010 A survey of graph edit distance. Pattern Anal. Appl. 13, 113–129. (10.1109/GlobalSIP.2013.6736904)

[32] Wallis W, Shoubridge P, Kraetz M, Ray D 2001 Graph distances using graph union. Pattern Recognit. Lett. 22, 701–704. (10.1016/S0167-8655(01)00022-8)

[33] Chowdhury S, Mémoli F 2017 Distances and isomorphism between networks and the stability of network invariants. (http://arxiv.org/abs/1708.04727)

[34] Carpi L, Rosso O, Saco P, Ravetti M 2011 Analyzing complex networks evolution through information theory quantifiers. Phys. Lett. A 375, 801–804. (10.1016/j.physleta.2010.12.038)

[35] Emmert-Streib F, Dehmer M, Shi Y 2016 Fifty years of graph matching, network alignment and network comparison. Inf. Sci. 346, 180–197. (10.1016/j.ins.2016.01.074)

[36] Meilǎ M 2007 Comparing clusterings-an information based distance. J. Multivariate Anal. 98, 873–895. (10.1016/j.jmva.2006.11.013)

[37] Jurman G, Visintainer R, Filosi M, Riccadonna S, Furlanello C 2015 The HIM glocal metric and kernel for network comparison and classification. In *Proc. of the 2015 IEEE Int. Conf. on Data Science and Advanced Analytics, DSAA*. 1–10 (10.1109/DSAA.2015.7344816).

[38] Mellor A, Grusovin A 2019 Graph comparison via the nonbacktracking spectrum. Phys. Rev. E 99, 052309 (10.1103/PhysRevE.99.052309) 31212453

[39] van Steen M 2010 Graph theory and complex networks. An Introduction. Amsterdam, The Netherlands: Maarten van Steen 978-90-815406-1-2.

[40] De Domenico M, Biamonte J 2016 Spectral entropies as information-theoretic tools for complex network comparison. Phys. Rev. X 6, 041062 (10.1103/PhysRevX.6.041062)

[41] Chen D, Shi DD, Qin M, Xu SM, Pan GJ 2018 Complex network comparison based on communicability sequence entropy. Phys. Rev. E 98, 012319 (10.1103/PhysRevE.98.012319) 30110844

[42] Hammond D, Gur Y, Johnson C 2013 Graph diffusion distance: a difference measure for weighted graphs based on the graph Laplacian exponential kernel. In *2013 IEEE Global Conf. on Signal and Information Processing, GlobalSIP 2013 - Proceedings*. 419–422 (10.1109/GlobalSIP.2013.6736904)

[43] Ipsen M, Mikhailov A 2002 Evolutionary reconstruction of networks. Phys. Rev. E 66, 046109 (10.1103/PhysRevE.66.046109) 12443261

[44] Molloy M, Reed B 1995 A critical point for random graphs with a given degree sequence. Random Struct. Algorithms 6, 161–180. (10.1002/rsa.3240060204)

[45] Del Genio CI, Gross T, Bassler KE 2011 All scale-free networks are sparse. Phys. Rev. Lett. 107, 1–4. (10.1103/PhysRevLett.107.178701) 22107590

[46] Newman MEJ 2005 Power laws, Pareto distributions and Zipf’s law. Contemp. Phys. 46, 323–351. (10.1080/00107510500052444)

[47] Krapivsky PL, Redner S, Leyvraz F 2000 Connectivity of growing random networks. Phys. Rev. Lett. 85, 4629 (10.1103/PhysRevLett.85.4629) 11082613

[48] McCabe S, Torres L, LaRock T, Haque SA, Yang C-H, Hartle H, Klein B 2020 netrd: A library for network reconstruction and graph distances. https://arxiv.org/abs/2010.16019.

