# Supplementary material for: Network comparison and the within-ensemble graph distance
Source: Proc Math Phys Eng Sci. 2020 Nov 4;476(2243):20190744. doi: 10.1098/rspa.2019.0744 (PMC7735290; doi:10.1098/rspa.2019.0744)
Supplement: Supplementary Information 1 [file rspa20190744supp1.pdf]

Supplementary Information for "Network comparison and the within-ensemble graph distance"

A. Within-ensemble graph distance as network size increases

In the main text figures, we plot the within-ensemble graph distances of networks with a fixed size. However, one important behavior of graph distance measures is how they change as networks increase in size.

As an example, the Jensen-Shannon divergence between the degree distributions (DJS) of two ER graphs will decrease as $n \to \infty$, since the empirical degree distributions get closer and closer to a binomial distribution. On the other hand, for graph distances that are explicitly accompanied by a size-normalizing term (e.g. HAM), we would expect that the mean within-ensemble graph distance does not change as network size increases.

In Figure 1, we show how the within-ensemble graph distance changes as n increases, both for a fixed density in $G_{(n,p)}$ as well as a fixed average degree in $G_{(n,\langle k \rangle)}$.

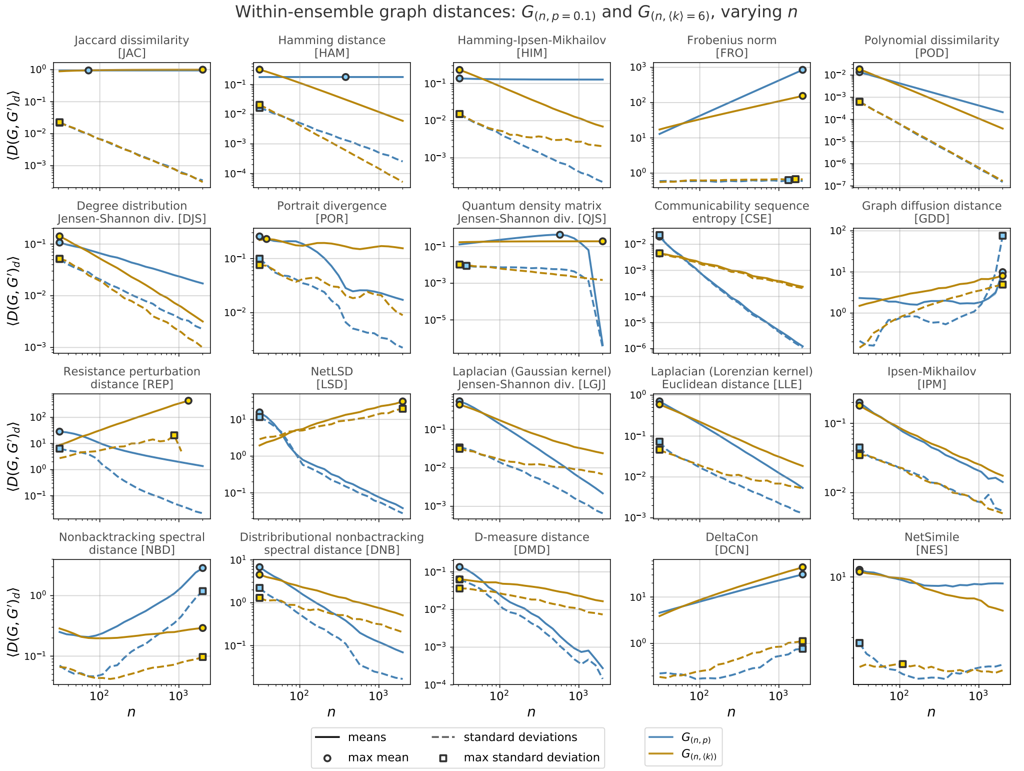

Figure 1. Mean and standard deviations of the within-ensemble distances for $G_{(n,p)}$ and $G_{(n,\langle k \rangle)}$ as n increases. Here, we generate pairs of ER networks with either a fixed density, p or with a fixed average degree, $\langle k \rangle$, as we increase the network size, n. In each subplot, the mean within-ensemble graph distance is plotted as a solid line with a shaded region around for the standard error ($\langle D \rangle \pm \sigma_{\langle D \rangle}$; note that in most subplots above, the standard error is too small to see), while the dashed lines are the standard deviations.

B. Descriptions of graph distance measures

Throughout the appendix, we assume graphs G and G' are undirected and unweighted so that the adjacency matrices are binary and symmetric. We first consider several projections for distances given a description which is the full adjacency sequence or matrix, followed by projections involving statistical and ad-hoc descriptions. The list of graph distances used in this work is {JAC, HAM, HIM, FRO, POD, DJS, POR, QJS, CSE, GDD, REP, LSD, LGJ, LLE, IPM, NBD, DNB, DMD, DCN, NES}.

(a) Jaccard Distance

The Jaccard measure is computed using the adjacency matrix $\psi_G = \mathbf{A} \in \{0,1\}^{n\times n}$. For two graphs vertex-labeled G and G',

$$D_{\texttt{JAC}}(G,G') = d_{\texttt{JAC}}(\mathbf{A},\mathbf{A}') = 1 - \frac{|\mathbf{S}|}{|\mathbf{T}|} \qquad \text{(A 1)}$$

where $S_{ij} = A_{ij}A'_{ij}$ represents the intersection of edge sets between graphs G and G', while $T_{ij} = S_{ij} + (1 - A'_{ij})A_{ij} + (1 - A_{ij})A'_{ij}$ represents the union of edge sets between graphs. Here, $|\mathbf{S}|$ is the sum over the S_{ij} and similarly for $|\mathbf{T}|$. The computational complexity of the Jaccard distance is $O(|E| + |E'|)$ when using unordered sets to get the union and intersection sets and their cardinality. This is what is done in the netrd package [1].

Since nearly empty graphs likely have nearly zero edges in common, the $|\mathbf{S}||\mathbf{T}|$ will be nearly zero for p close to 0, so that $d_{\texttt{JAC}}$ approaches 1 at low p.

(b) Hamming Distance

Similarly, the Hamming measure may also be computed using the adjacency matrix $\mathbf{A} \in \{0,1\}^{n\times n}$. For two vertex-labeled graphs G and G', the Hamming distance counts the number of elementwise differences between $\psi_G = \mathbf{A}$ and $\psi_{G'} = \mathbf{A}'$:

$$D_{\texttt{HAM}}(G,G') := \frac{1}{\binom{n}{2}} \sum_{1\le i<j\le n} |A_{ij} - A'_{ij}|. \qquad \text{(A 2)}$$

The computational complexity of the Hamming distance is $O(n^2)$ if one compares the elements A_{ij} for each node-pair ij. This is what is used in the netrd package [1]. For sparse graphs, one could use unordered sets of edges to only compute the distance on edges (i,j) in the union set $E \cup E'$, leading to a computational complexity of $O(|E| + |E'|)$.

(c) Frobenius

The Frobenius distance $d_{\texttt{FRO}}$ is simply the norm of matrices, so that:

$$D_{\texttt{FRO}}(G,G') := \sqrt{\sum_{i,j} |A_{ij} - A'_{ij}|^2} \qquad \text{(A 3)}$$

Note that for binary adjacency matrices, $|A_{ij} - A'_{ij}|^2 = |A_{ij} - A'_{ij}|$, and $A_{ii} = A'_{ii} = 0\,\forall i$ given that there are no self-loops. Note that, because the distance operates on the adjacency matrices directly, it implicitly assumes the graphs are vertex-labeled. FRO has the same computational complexity as the Hamming distance due to their similarity. It is $O(n^2)$ if one compares all entries, as is in the netrd package, but it could be improved to $O(|E| + |E'|)$.

(d) Polynomial Dissimilarity

The polynomial dissimilarity, POD, between two unweighted, vertex-labeled graphs is based on the eigenvalue decompositions of the two adjacency matrices of the graphs, G and G' [2].

To compute the polynomial dissimilarity between two graphs, first decompose A as $Q_A \Lambda_A Q_A^T$, where Q_A is an orthogonal matrix and Λ_A is the diagonal matrix of eigenvalues. Second, construct vectors $P(A)$ and $P(A')$ for each graph, where $P(A) = Q_A W_A Q_A^T$ and $W_A = \Lambda_A + \frac{1}{(n-1)^\alpha}\Lambda_A^2 + \ldots + \frac{1}{(n-1)^{\alpha(K-1)}}\Lambda_A^K$.

The polynomial dissimilarity, then, is calculated as the Frobenius norm between $P(A)$ and $P(A')$

$$D_{\texttt{POD}}(G, G') = \frac{1}{n^2}||P(A) - P(A')||\,. \tag{A 4}$$

In this work, we consider a default value of $K = 5$ in order to accommodate potentially informative higher-order interactions in each of the graphs. Here, $\alpha = 1$ by default, though in [2], $\alpha = 0.9$ is commonly considered.

The computational complexity of POD is $O(n^3)$ in practice, which arises from it requiring two $n \times n$ matrix eigendecompositions, which is $O(n^3)$ for general matrices and a method based on the QR algorithm [3], as used in the `netrd` package. Note that recent techniques based on message-passing can give fast and exact results for sparse networks with short loops in $O(n \log n)$ [4] and could be used to reduce the computational complexity of spectral graph distances.

(e) Degree Distribution Jensen-Shannon Divergence

A simple graph distance measure is the Jensen-Shannon divergence [5] between the empirical degree distributions of two graphs. In this case for an n-node graph G the descriptor ψ_G is the empirical degree distribution encoded in the set of numbers $\{p_k(G)\}_{k\geq 0} := \mathbf{p}$ given by $p_k(G) := n_k(G)/n$, where $n_k(G) = \sum_{i=1}^{n} \mathbf{1}\{k_i = k\}$, with $\mathbf{1}\{\cdot\}$ being the indicator function and $k_i = \sum_{j=1}^{n} A_{ij}$ being the degree of node i in terms of the adjacency matrix $\mathbf{A}$ of G. The Jensen-Shannon divergence between two such distributions [6] is the *degree* Jensen-Shannon divergence or **DJS** distance between the graphs:

$$D_{\texttt{DJS}}(G, G') = H\left[\mathbf{p}_+\right] - \frac{1}{2}\left(H[\mathbf{p}] + H[\mathbf{p}']\right), \tag{A 5}$$

where $\mathbf{p}_+ = \{(p_k + p'_k)/2\}_{k\geq 0}$ is a mixture distribution and $H[\mathbf{p}] = -\sum_k p_k \ln p_k$ is the Shannon entropy.

The computational complexity of DJS is $O(n)$, which arises from computing two degree distributions (which is $O(n)$) and then comparing them (which is $O(k_+)$, with $k_+ < n$ being the maximum degree in either network).

(f) Portrait Divergence

The portrait divergence, POR, compares using the JSD a description for each of two graphs called their *network portrait* [7,8]. The network portrait is a matrix B with elements B_{lk} such that

$$B_{lk} \equiv \text{number of nodes with } k \text{ nodes at distance } l. \tag{A 6}$$

Alternatively stated, B_{lk} is the kth entry of the empirical histogram of l-th neighborhood sizes. These elements are computed using a breadth-first search or similar method. The portrait divergence of G and G' is the JSD of probability distributions associated with their portraits, B and B' [8]. Note that each row in B can be interpreted as the probability distribution that there will be k nodes at a distance of l away from a randomly chosen node such that:

$$P(k|l) = \frac{B_{l,k}}{N} \tag{A 7}$$

which can be normalized of the number of paths of length l such that the probability distribution is the probability that two randomly selected nodes are at a distance l away from each other:

$$P(l) = \frac{\sum_{k=0}^{n} kB_{l,k}}{\sum_c n_c^2} \tag{A 8}$$

where n_c is the number of nodes within a connected component, c. The joint probability of choosing a pair of nodes at a distance, l, away from each other *and* that one node has k nodes in total at distance, l, away is:

$$P(k,l) = P(k|l)P(l) = \left(\frac{\sum_{k'=0}^{n} k' B_{l,k'}}{n}\right) \frac{B_{l,k}}{\sum_c n_c^2} \tag{A 9}$$

There is now a $P_B(k,l)$ and $P_{B'}(k,l)$ for each portrait, B and B', as well as a "mixed" distribution for both, which is specified as $P^* = \frac{1}{2}(P_B(k,l) + P_{B'}(k,l))$. The portrait divergence between G and G' is the JSD between their portraits as follows

$$\begin{aligned} D_{\texttt{POR}}(G,G') =& JSD(P_B(k,l), P_{B'}(k,l)) \\ =& \frac{1}{2}\big(D_{KL}(P_B(k,l), P^*)+ \\ & D_{KL}(P_{B'}(k,l), P^*)\big) \end{aligned} \tag{A 10}$$

where D_{KL} is the Kullback-Leibler divergence. Note that $\sqrt{D_{\texttt{POR}}}$ satisfies the properties of a metric (satisfies the triangle inequality, is positive-definite, symmetric) [9].

The computational complexity of POR is $O(n(n+|E|)\log n)$, which comes from the requirement of computing shortest paths between all pairs of nodes in the network. In our implementation, computing the shortest path between a source and all nodes is done with the Dijkstra's algorithm with a binary heap, which takes $O((n+|E|)\log n)$ operations in the worst case. Constructing the portrait and calculating the JSD between the associated distributions has a lower computational complexity.

(g) Quantum Spectral Jensen-Shannon Divergence

This method compares graphs via the Jensen-Shannon divergence (JSD) between probability distributions associated with density matrices of two graphs G and G' [10–12], denoted ρ and ρ' respectively, defined by

$$\rho = \frac{e^{-\beta \mathbf{L}(G)}}{Z} \tag{A 11}$$

where $\mathbf{L}(G)$ is the Laplacian matrix of graph G, and constant $Z \equiv \sum_{i=1}^{n} e^{-\beta\lambda_i(\mathbf{L})}$, with $\lambda_i(\mathbf{L})$ being the ith eigenvalue of $\mathbf{L}$. Description-distance pair (ρ, JSD) yields the "Quantum Spectral Jensen-Shannon Divergence" (QJS) [13], which compares two graphs by the entropy of the eigenvalue spectra of their density matrices ρ. Treating the spectrum $\{\lambda_i\}_{i=1}^{n}$ as a normalized probability distribution, the spectral Rényi entropy of order q is given by

$$S_q = \frac{1}{1-q}\log_2 \sum_{i=1}^{n} \lambda_i(\rho)^q, \tag{A 12}$$

which, if $q=1$, reduces to the Von Neumann entropy:

$$S_1 = -\sum_{i=1}^{n} \lambda_i(\rho)\log_2 \lambda_i(\rho). \tag{A 13}$$

The QJS distance between two graphs is defined to be:

$$D_{\texttt{QJS}}(G,G') = S_q\left(\frac{\rho+\rho'}{2}\right) - \frac{1}{2}[S_q(\rho) + S_q(\rho')]. \tag{A 14}$$

For default parameter values, we use $\beta = 0.1$ and $q = 1.0$, based on the explanations in [13]. QJS requires computation of Laplacian matrix spectra of two graphs, and comparison thereof, which yields a computational complexity of $O(n^3)$ (see Appendix (d)).

(h) Communicability Sequence Entropy Divergence

The communicability sequence entropy divergence CSE between two graphs, G and G', is the JSD between the communicability distributions of G and G' [14]. In order to have a communicability distribution, we first construct the communicability matrix, which is an $n \times n$ matrix corresponding to the *communicability* between two nodes, v_i and v_j.

$$C = e^A = \sum_{k=0}^{\infty} \frac{1}{k!} A^k \tag{A 15}$$

In other words, the communicability matrix, C, is computed as a matrix exponentiation of the adjacency matrix. The elements $C_{ij}, i \leq j$, are stored in a vector (of length $\binom{n}{2}$) and normalized to create the communicability sequence, P and P', for each graph. The Shannon entropy of P is $H[P] = -\sum_{i=1}^{M} P_i \log_2 P_i$, and the communicability sequence entropy divergence is calculated as the JSD between P and P', where M is the mixed sequence of P and P'.

$$D_{\texttt{CSE}}(G, G') = JSD(P, P') = H[M] - \frac{1}{2}(H[P] + H[P']) \,. \tag{A 16}$$

The computational complexity of CSE is $O(n^3)$, with the computationally intensive step being to compute the exponential of both adjacency matrices A and A'. Our implementation uses Padé approximants through the SciPy package to perform this step, which takes $O(n^3)$ operations to get an approximation [15].

(i) Graph Diffusion Distance

The graph diffusion distance [16] GDD between two graphs, G and G', is a distance measure based on the notion of *flow* within each graph. As such, this measure uses the unnormalized Laplacian matrices of both graphs, $\mathbf{L}$ and $\mathbf{L}'$, and uses them to construct time-varying Laplacian exponential diffusion kernels, $e^{-t\mathbf{L}}$ and $e^{-t\mathbf{L}'}$, by effectively simulating a diffusion process for t timesteps (as a default, $t = 1000$), creating a column vector of node-level activity at each timestep.

The distance $d_{\texttt{GDD}}(G, G')$ is defined as the Frobenius norm between the two diffusion kernels at the timestep t^* where the two kernels are maximally different.

$$D_{\texttt{GDD}}(G, G') = \sqrt{||e^{-t^*\mathbf{L}} - e^{-t^*\mathbf{L}'}||} \tag{A 17}$$

The computational complexity is $O(n^3)$ since a spectral decomposition of the Laplacian matrices is used (see Appendix (d)).

(j) Resistance Perturbation Distance

The resistance perturbation distance RES between two vertex-labeled graphs, G and G', is the p-norm of the difference between two graph resistance matrices [17]. The resistance perturbation distance changes if either graph is relabeled (it is not invariant under graph isomorphism), so node labels should be consistent between the two graphs being compared. The distance is not normalized.

The resistance matrix of a graph G is calculated as

$$R = \text{diag}(\mathcal{L})\mathbf{1}^T + \mathbf{1}\text{diag}(\mathcal{L})^T - 2\mathcal{L}, \tag{A 18}$$

where $\mathcal{L}$ is the Moore-Penrose pseudoinverse of the Laplacian of G.

The resistance perturbation graph distance of G and G' is calculated as the p-norm (the pth root of the sum of the pth powers of elements) of the difference in their resistance matrices, $R^{(1)}$

and $R^{(2)}$

$$D_{\texttt{REP}}(G,G') = \left[\sum_{i,j \in V} |R_{i,j} - R'_{i,j}|^p \right]^{1/p} . \tag{A 19}$$

The default value chosen in experiments is $p = 2$. The computational complexity of RES is $O(n^3)$ for our implementation, since we need to compute the Moore-Penrose pseudoinverse of the Laplacian matrix of both graphs, which is $O(n^3)$. Note that low-rank approximations can be used to reduce the computational complexity [17].

(k) NetLSD

The NetLSD distance LSD between two graphs, G and G', is the Frobenius norm between the heat trace signatures of the normalized Laplacians $\mathbf{L}$ and $\mathbf{L}'$ [18]. The heat kernel matrix is calculated as

$$H_t = e^{-t\mathbf{L}} = \sum_{j=1}^{n} e^{-t\lambda_j} \phi_j \phi_j^T . \tag{A 20}$$

The ij-th element of H_t contains the amount of heat transferred from node v_i to node v_j at time t (default of 256 log-spaced time intervals between 10^{-2} and 10^2). From the heat kernel matrix H_t, the *heat trace*, h_t is defined as

$$h_t = \text{Tr}(H_t) = \sum_{j=1}^{n} e^{-t\lambda_j} . \tag{A 21}$$

The *heat trace signature* of graph G is the set $\{h_t\}_{t \geq 1}$. Upon computing heat trace signatures of both G and G', they are compared via a Frobenius norm

$$D_{\texttt{LSD}}(G,G') = d_{\texttt{FRO}} \left(\{h_t\}_{t \geq 0}, \{h'_t\}_{t \geq 0} \right) . \tag{A 22}$$

The computational complexity of LSD is $O(n^3)$ due to the spectral decomposition of the Laplacian matrices of both graphs (see Appendix (d)).

(l) Laplacian Spectrum Distances

Many distances between two graphs, G and G', use a direct comparison of their Laplacian spectrum. For all the methods below, we use the eigenvalues $\{\lambda_1 = 0 \leq \lambda_2 \leq \cdots \leq \lambda_n\}$ of the normalized Laplacian matrices $\mathbf{L}$ and $\mathbf{L}'$. To perform the comparison, a subset of the whole spectrum can be used, e.g. the k smallest [19] or largest [12,20] in magnitude. Unless specified, we used all eigenvalues for comparison ($k = n$).

The distances compare the continuous spectra $\rho(\lambda)$ and $\rho'(\lambda)$ associated with the graph G and G'. A continuous spectrum is obtained by the convolution of the discrete spectrum $\sum_i \delta(\lambda - \lambda_i)$ with a kernel $g(\lambda, \lambda^*)$

$$\rho(\lambda) = \frac{1}{Z} \sum_{i=1}^{n} \int_0^2 g(\lambda, \lambda^*) \delta(\lambda^* - \lambda_i) \mathrm{d}\lambda^* , \tag{A 23}$$

where Z is a normalization factor. Different types of distribution can be used for the kernel, for instance a Lorentzian distribution [21]

$$g(\lambda, \lambda^*) = \frac{\gamma}{\pi[\gamma^2 + (\lambda - \lambda^*)^2]} , \tag{A 24}$$

or a Normal distribution

$$g(\lambda, \lambda^*) = \frac{\exp[-(\lambda - \lambda^*)^2 / 2\sigma^2]}{\sqrt{2\pi\sigma^2}} . \tag{A 25}$$

Different types of metrics can then be used to compare the spectra, such as the Euclidean metric

$$d(\rho, \rho') = \sqrt{\int_0^2 [\rho(\lambda) - \rho'(\lambda)]^2 \mathrm{d}\lambda} , \tag{A 26}$$

or the square root of the JSD $d(\rho, \rho') = \sqrt{JSD(\rho, \rho')}$, written as

$$JSD(\rho, \rho') = \frac{1}{2} D_{KL}(\rho || \bar{\rho}) + \frac{1}{2} D_{KL}(\rho' || \bar{\rho}) \tag{A 27}$$

where $\bar{\rho} = (\rho + \rho')/2$. Various combination of kernels and metrics yield the following distinct distance measures:

- Laplacian spectrum: Gaussian kernel, JSD distance `LGJ`
- Laplacian spectrum: Lorenzian kernel, Euclidean distance `LLE`

For both kernels, we use a *half width at half maximum* of 0.011775 (which means the standard deviation for the Gaussian kernel is ≈ 0.01).

While we only focus on the two specific distances above, we note again that there is a world of possible combinations of descriptor-distance pairs to possibly use for comparing graphs. We selected the two above because their within-ensemble graph distance curves differed the most (e.g. as opposed to including Gaussian kernel / Euclidean distance or Lorenzian kernel / JSD). The computational complexity of this suite of graph distances is $O(n^3)$ due to the spectral decomposition of the Laplacian matrices of both graphs (see Appendix (d)).

(m) Ipsen-Mikhailov

The Ipsen-Mikhailov distance [21] `IPM` between two graphs, G and G', is a spectral comparison of their Laplacian matrices, $\mathbf{L}$ and $\mathbf{L}'$. This approach treats the set of nodes in G and G' as molecules with an elastic connection between them, which casts the distance measurement between G and G' as the solution to a set of differential equations between the vibrational frequencies between the nodes. The vibrational frequencies, ω_i, of each node in G is related to the eigenvalues, λ, of $\mathbf{L}$ such that $\lambda_i = \omega_i^2$.

With this, one can construct a spectral density for each graph as a sum of Lorenz distributions as follows

$$\rho(\omega) = \frac{1}{Z} \sum_{i=1}^{n-1} \frac{\gamma}{(\omega - \omega_i)^2 + \gamma^2} \tag{A 28}$$

where Z is a normalization term, and γ is a fixed scaling term that controls the width of the Lorenz distributions (as in [21], we use $\gamma = 0.08$ as a default). The distance between G and G' is then calculated as

$$D_{\texttt{IPM}}(G, G') = d(\rho, \rho') = \sqrt{\int_0^\infty [\rho(\omega) - \rho'(\omega)]^2 d\omega} \tag{A 29}$$

The computational complexity of `IPM` is $O(n^3)$ due to the spectral decomposition of the Laplacian matrices of both graphs (see Appendix (d)).

(n) Hamming-Ipsen-Mikhailov

The Hamming-Ipsen-Mikhailov distance `HIM` between two vertex-labeled graphs, G and G' is expressed as a weighted combination of the `IPM` (Section (m)) distance and a normalized `HAM` (Section (b)) distance [22]. The parameter γ for the `IPM` is fixed such that $D_{\texttt{IPM}}(\mathcal{E}_n, \mathcal{F}_n) = 1$, where $\mathcal{E}_n$ and $\mathcal{F}_n$ are the empty and complete graphs of n nodes. The `HIM` distance is defined as follows

$$D_{\texttt{HIM}}(G, G') = \frac{1}{\sqrt{1+\xi}} \sqrt{D_{\texttt{IPM}}(G, G')^2 + \xi D_{\texttt{HAM}}(G, G')^2} \tag{A 30}$$

We default to $\xi = 1$, as in [22]. The computational complexity of `HIM` is $O(n^3)$, with the computationally intensive part being the computation of the `IPM` distance.

(o) Non-backtracking Spectral Distance

The non-backtracking spectral distance NBD between two graphs, G and G', is a method that compares the eigenvalues of the non-backtracking matrix of each graph, $\mathcal{B}$ and $\mathcal{B}'$ [23]. This distance is based on the length spectrum and the set of non-backtracking cycles of a graph (i.e., a closed walk that does not immediately return to the node from which it left) and is calculated as the earth mover's distance (EMD) between the eigenvalues of $\mathcal{B}$ and $\mathcal{B}'$. The eigenvalues of $\mathcal{B}$ and $\mathcal{B}'$ are expressed as $\lambda_k = a_k + ib_k$ and $\lambda'_k = a'_k + ib'_k$, respectively, and $EMD(\lambda_{\mathcal{B}}, \lambda_{\mathcal{B}'})$ is the solution to an optimization problem finding the minimum amount of work required to move the coordinates of λ to the positions of λ'.

$$D_{\texttt{NBD}}(G, G') = EMD(\lambda_{\mathcal{B}}, \lambda_{\mathcal{B}'})\,. \tag{A 31}$$

Note that the Ihara determinant formula can be used to obtain the non-backtracking eigenvalues different from ± 1 using a $2n \times 2n$ matrix [23].

If one uses the whole non-backtracking spectrums to compute the distance, the computational complexity would be $O(n^3)$ [23]. Instead of using the whole spectrum of the non-backtracking matrices, for graph G we compute only the r eigenvalues larger in magnitude than $\sqrt{\lambda_1}$, where λ_1 is the largest eigenvalue of $\mathcal{B}$ [23].

The computational complexity of our implementation of NBD is $O(\max(r, r')n^2)$ for general graphs, where r and r' are the number of eigenvalues larger in magnitude than $\sqrt{\lambda_1}$ and $\sqrt{\lambda'_1}$, respectively for graph G and G'. To compute these eigenvalues, an implicitly restarted Arnoldi method is used. For sparse graphs the computation is even more efficient.

(p) Distributional Non-backtracking Distance

Similar to the NBD distance [24], the DNB distance leverages spectral properties of the non-backtracking matrices, $\mathcal{B}$ and $\mathcal{B}'$, of two graphs, G and G', in order to calculate their dissimilarity.

Unlike the NBD distance, the DNB involves a comparison of the (re-scaled) distribution of eigenvalues of $\mathcal{B}$ and $\mathcal{B}'$, which are then compared using either the Euclidean distance or the Chebyshev distance (here, we use the Euclidean distance). We also use the whole spectrum for this distance. Therefore, the computational complexity of DNB is $O(n^3)$ due to the spectral decomposition of the two $2n \times 2n$ matrices (see Appendix (o)).

(q) *D*-measure Distance

The D-measure distance [25] DMD between two graphs, G and G', involves a combination of three properties from the two graphs to be compared, G and G': the *network node dispersion* (NND), *the node distance distribution* (μ), and the *α-centrality* (α) for each graph. For a full explanation and justification for each of the components involved in this distance, we refer the reader to the original article [25], but we will briefly summarize it below.

In order to compute the NND of a graph, each node, v_i, is assigned a probability vector, $\mathbf{P}_i$, with elements that are the fraction of nodes that are connected to v_i at each distance $j \le d$, where d is the diameter of the network. The NND, then, is defined as

$$NND(G) = \frac{JSD\big(\mathbf{P}_1, \mathbf{P}_2, ..., \mathbf{P}_n\big)}{\log(d+1)} \tag{A 32}$$

where $JSD\big(\mathbf{P}_1, \mathbf{P}_2, ..., \mathbf{P}_n\big)$ is the Jensen-Shannon divergence of each $\mathbf{P}_i$ from the whole network's average node-distance distribution at every distance j, which we will denote μ_j. The average μ_j for all distances $j \le d$ in a graph, G, we will denote μ_G.

The final step before the calculation of the D-measure distance is to find the α-centrality [26] of each network, G and G', as well as the α-centrality of the *complement* of each network, G^c and $G^{c\prime}$. The α-centralities of the original networks are denoted $P_{\alpha G}$ and $P_{\alpha G'}$, while the α-centralities of their complements are $P_{\alpha G^c}$ and $P_{\alpha G^{c\prime}}$.

Ultimately, the D-measure distance, D_{DMD}, between two graphs is as follows:

$$D_{\mathrm{DMD}}(G,G') = w_1\sqrt{\frac{JSD(\mu_G,\mu_{G'})}{\log(2)}} +$$
$$w_2\left|\sqrt{NND(G)} - \sqrt{NND(G')}\right| +$$
$$\frac{w_3}{2}\left(\sqrt{\frac{JSD(P_{\alpha G},P_{\alpha G'})}{\log(2)}} + \sqrt{\frac{JSD(P_{\alpha G^c},P_{\alpha G^{c\prime}})}{\log(2)}}\right) \quad \text{(A 33)}$$

where $w_1 + w_2 + w_3$ must equal 1.0. To calculate the final distance value, we adopt the convention used in [25] such that $w_1 = 0.45$, $w_2 = 0.45$, $w_3 = 0.1$.

According to Ref. [25], the computational complexity of DMD is $O(|E| + n\log n)$. However, one needs to compute all shortest paths between all nodes, which suggest a more computationally intensive calculation. We rather have a computational complexity of $O(n(n + |E|)\log n)$ with our implementation using Dijkstra algorithm with a binary heap (see Appendix (f)).

(r) DeltaCon

The DeltaCon distance DCN between two graphs, G and G', is the Matusita distance between the *affinity* matrices, S and S', of G and G'. The affinity matrices are constructed using Fast Belief Propagation, which is expressed as

$$[\mathbf{I} + \epsilon^2\mathbf{D} - \epsilon\mathbf{A}]\vec{s_i} = \vec{e_i} \quad \text{(A 34)}$$

where $\mathbf{I}$ is the $n \times n$ identity matrix, $\mathbf{D}$ is the diagonal degree matrix, $\mathbf{A}$ is the adjacency matrix, $\vec{e_i}$ is a vector indicating the initial node v_i from which a random walk process is initiated, and $\vec{s_i}$ is a column vector consisting of s_{ij}, which is the affinity of node v_j with respect to node v_i. The affinity matrices, S and S', are defined as $S = [\mathbf{I} + \epsilon^2\mathbf{D} - \epsilon\mathbf{A}]^{-1}$. The distance between G and G' according to DeltaCon is as follows

$$D_{\mathrm{DCN}}(G,G') = d(S,S') = \sqrt{\sum_{i=1}^{n}\sum_{j=1}^{n}\left(\sqrt{s_{ij}} - \sqrt{s'_{ij}}\right)^2} \quad \text{(A 35)}$$

The computational complexity of our implementation of DCN is $O(n^3)$ since we obtain S by matrix inversion directly. However, note that it is possible to improve the algorithm and have an $O(n^2)$ computational complexity using a power method or even $O(|E|)$ by approximating the distance [27].

(s) NetSimile

NetSimile NES is a method for comparing two graphs, G and G', that is based on statistical features of the two graphs. It is invariant to graph labels and is able to compare graphs of different sizes [28]. It is calculated as the Canberera distance between the 7×5 feature matrix, $\mathbf{p}$ and $\mathbf{p}'$, of each graph. To construct the $\mathbf{p}$ and $\mathbf{p}'$ feature matrices, first a $7 \times n$ matrix is constructed for each, with each column, j, consisting of the following seven node-level quantities:

(i) degree, $k_j = \sum_j A_{ij}$
(ii) clustering coefficient, $c_j = (A^3)_{jj}/\binom{k_j}{2}$
(iii) average neighbor degree $k_j^{(nn)} = \frac{1}{k_j}\sum_i k_i A_{ij}$.
(iv) average clustering coefficient of the nodes in the ego network $c_j^{(ego)} = \sum_i c_i A_{ij}$
(v) number of edges within the ego network $T_j = \sum_{l,m} A_{jl}A_{lm}A_{mj}$
(vi) number of outgoing edges from the ego network $O_j = \sum_i A_{ij}k_i - T_j = k_j k_j^{(nn)} - T_j$
(vii) number of neighbors of the ego network $nn_j^{(ego)} = \sum_i \mathbf{1}_{\{\exists l \in \mathcal{N}_j : i \sim l, i \not\sim j\}}$

These features are then summarized into $\mathbf{p}$ and $\mathbf{p}'$, which are 7×5 signature vectors consisting of the median, mean, standard deviation, skewness, and kurtosis of each feature. NetSimile uses the Canberra distance to arrive at a final scalar distance.

$$D_{\texttt{NSE}}(G, G') = d(\mathbf{p}, \mathbf{p}') = \sum_{i=1}^{n} \frac{|p_i - p_i'|}{|p_i| + |p_i'|} \tag{A 36}$$

The computational complexity of NES depends on two parts : features extraction and features aggregation. Features are all locally defined, hence their extraction will take $O(qn)$ where q is the average degree of a node when selecting a random edge and choosing an endpoint [29]. Feature aggregation is $O(n \ln n)$ [28], hence the overall complexity is $O(qn + n \log n)$.

C. Analytical derivation of within-ensemble graph distances

(a) Jaccard Distance

We can directly calculate $\langle d_{\texttt{JAC}}(\mathbf{A}, \mathbf{A}')\rangle_{G(n,p)}$, the expected Jaccard distance among two graphs sampled from $G_{(n,p)}$. Both $|\mathbf{T}|$ and $|\mathbf{S}|$ are distributed binomially, as they are the sum of $\binom{n}{2}$ Bernoulli values arising with probability p^2 and $2p(1-p)+p^2$, respectively. Since binomial distributions are sharply peaked (for large values of n), we can approximate the expected value of the ratio $|\mathbf{S}|/|\mathbf{T}|$ by the ratio of the expected values of $|\mathbf{S}|$ and $|\mathbf{T}|$. Thus we have,

$$\begin{aligned}
\langle D_{\texttt{JAC}}(\mathbf{A}, \mathbf{A}')\rangle_{G(n,p)} &= 1 - \left\langle \frac{|\mathbf{S}|}{|\mathbf{T}|} \right\rangle \\
&\approx 1 - \frac{\langle|\mathbf{S}|\rangle}{\langle|\mathbf{T}|\rangle} \\
&= 1 - \frac{p^2\binom{n}{2}}{(2p(1-p)+p^2)\binom{n}{2}} \\
&= \frac{1-p}{1-\frac{p}{2}}
\end{aligned} \tag{A 1}$$

which agrees precisely with simulations. Note, in the limit $p \approx 1$, we have by Taylor expansion,

$$\begin{aligned}
\langle D_{\texttt{JAC}}(\mathbf{A}, \mathbf{A}')\rangle_{G(n,p\approx 1)} &= 1 - \left\langle \frac{|\mathbf{S}|}{|\mathbf{T}|} \right\rangle\Bigg|_{p\approx 1} \\
&= \frac{1-p}{1-\frac{p}{2}}\Bigg|_{p=1} + (p-1)\frac{d}{dp}\left(\frac{1-p}{1-\frac{p}{2}}\right)\Bigg|_{p=1} + \ldots \\
&= 0 + (p-1)\left(\frac{-1}{1-\frac{p}{2}} + \frac{-(1-p)(-\frac{1}{2})}{(1-\frac{p}{2})^2}\right)\Bigg|_{p=1} + \ldots \\
&= (p-1)\left(\frac{-1}{1-\frac{1}{2}} + 0\right) + \ldots \\
&= 2(1-p) + \ldots,
\end{aligned} \tag{A 2}$$

Similarly—as we show in SI (b)—the Hamming distance ($d_{\texttt{HAM}}$) behaves in this region as

$$\begin{aligned}
\langle D_{\texttt{HAM}}(\mathbf{A}, \mathbf{A}')\rangle_{G(n,p\approx 1)} &= 2p(1-p)|_{p=1} + (p-1)\left(2(1-p) - 2p\right)|_{p=1} + \ldots \\
&= 0 + (p-1)(0-2) + \ldots \\
&= 2(1-p) + \ldots,
\end{aligned} \tag{A 3}$$

which is exactly the same. Indeed, we observe this equivalence in Figure 1 of the main text in the region $p \approx 1$. This finding makes intuitive sense because in the region $p \approx 1$, the "union graph",

$\mathbf{T}$, is likely an essentially complete graph, and $d_{\texttt{JAC}}$ simply measures the fraction of edges/non-edges that are not in agreement between G and G', which is precisely what $d_{\texttt{HAM}}$ does for all p given an adjacency description.

(b) Hamming Distance

The Hamming measure is simply the fraction of mismatched entries between A and A'. Due to this simplicity, we again can analytically predict the mean within-ensemble graph distance for graphs sampled from $G_{(n,p)}$:

$$\begin{aligned}\langle D_{\texttt{HAM}}(\mathbf{A},\mathbf{A}')\rangle_{G(n,p)} &= \frac{1}{\binom{n}{2}} \sum_{1\le i<j\le n} \mathbb{P}(|A_{ij}-A'_{ij}|=1) \\ &= 2p(1-p).\end{aligned} \tag{A 4}$$

The function $2p(1-p)$ is n-independent, and has a maximum at $p=\frac{1}{2}$; simulations are matched by it precisely. Interestingly, while this calculation was done for $G_{(n,p)}$, the results in Figure 1 of the main text shows an equivalent result for RGGs of the same edge-density p.

(c) Frobenius

As a back-of-the envelope-calculation, note that the sum of elementwise differences is binomially distributed with mean $\langle \sum_{i,j} |A_{ij}-A'_{ij}|\rangle = n(n-1)2p(1-p)$. Using sharply-peakedness, we can thus state approximately,

$$\begin{aligned}\langle D_{\texttt{FRO}}(\mathbf{A},\mathbf{A}')\rangle_{G(n,p)} &= \left\langle \sqrt{\sum_{i,j}|A_{ij}-A'_{ij}|^2} \right\rangle \\ &\approx \sqrt{\left\langle \sum_{i,j}|A_{ij}-A'_{ij}| \right\rangle} \\ &\simeq n\sqrt{2p(1-p)},\end{aligned} \tag{A 5}$$

which exhibits a maximum at $p=\frac{1}{2}$ for any given n, but grows linearly with n, the latter two observations are qualitatively born out in simulations.

References

1. McCabe, S., Torres, L., LaRock, T., Haque, S., Yang, C-H., Hartle, H., Klein, B. 2019 `netrd`: Network reconstruction and graph distance measures in python. (`https://github.com/netsiphd/netrd`).
2. Donnat, C., Holmes, S. 2018 Tracking network dynamics: A survey using graph distances. *The Annals of Applied Statistics*. **12**.2, 971–1012. (doi:10.1214/18-AOAS1176).
3. Pan, V.Y., Chen, Z.Q. 1999 The complexity of the matrix eigenproblem. in *Proceedings of the Thirty-First Annual ACM Symposium on Theory of Computing*. Association for Computing Machinery. 507–516. (doi:10.1145/301250.301389).
4. Cantwell, G.T., Newman, M.E.J. 2019 Message passing on networks with loops. *Proceedings of the National Academy of Sciences*. **116**.47, 23398–23403. (doi:10.1073/pnas.1914893116).
5. Lin, J. 1991 Divergence measures based on the Shannon entropy. *IEEE Transactions on Information Theory*. **37**.1, 145–151. (doi:10.1109/18.61115).
6. Carpi, L., Rosso, O., Saco, P., Ravetti, M. 2011 Analyzing complex networks evolution through Information Theory quantifiers. *Physics Letters A*. **375**.4, 801–804. (doi:10.1016/j.physleta.2010.12.038).
7. Bagrow, J., Bollt, E., Skufca, J. D., Ben-Avraham, D. 2008 Portraits of complex networks. *Europhysics Letters*. **81**.6, 1–15. (doi:10.1209/0295-5075/81/68004).

8. Bagrow, J., Bollt, E. 2019 An information-theoretic, all-scales approach to comparing networks. *Applied Network Science*. **45**.4, 1–15. (doi:10.1007/s41109-019-0156-x).
9. Sutherland, W.A. 2009 *Introduction to Metric and Topological Spaces*. Oxford University Press. 978-0199563081.
10. Bai, L., Rossi, L., Torsello, A., Hancock, E.R. 2015 A quantum Jensen-Shannon graph kernel for unattributed graphs. *Pattern Recognition*. **48**.2, 344–355. (doi:10.1016/j.patcog.2014.03.028).
11. Rossi, L., Torsello, A., Hancock, E.R., Wilson, R.C. 2013. Characterizing graph symmetries through quantum Jensen-Shannon divergence. *Physical Review E*. **88**.3, 032806. (doi:10.1103/PhysRevE.88.032806).
12. Masuda, N., Holme, P. 2019 Detecting sequences of system states in temporal networks. *Scientific Reports*. **9**.1, 1–11. (doi:10.1038/s41598-018-37534-2).
13. De Domenico, M., Biamonte, J. 2016 Spectral entropies as information-theoretic tools for complex network comparison. *Physical Review X*. **6**.4, 041062. (doi:10.1103/PhysRevX.6.041062).
14. Chen, D., Shi, D.D., Qin, M., Xu, S.M., Pan, G.J. 2018 Complex network comparison based on communicability sequence entropy. *Physical Review E*. **98**.1, 1–8. (doi:10.1103/PhysRevE.98.012319).
15. Moler, C., Van Loan, C. 2003 Nineteen dubious ways to compute the exponential of a matrix, twenty-five years later. *SIAM Review*. **45**.1, 3–49. (doi:10.1137/S00361445024180).
16. Hammond, D., Gur, Y., Johnson, C. 2013 Graph diffusion distance: A difference measure for weighted graphs based on the graph Laplacian exponential kernel. *2013 IEEE Global Conference on Signal and Information Processing, GlobalSIP 2013 - Proceedings*. 419–422. (doi:10.1109/GlobalSIP.2013.6736904).
17. Monnig, N., Meyer, F. 2018 The resistance perturbation distance: A metric for the analysis of dynamic networks. *Discrete Applied Mathematics*. **236**, 347–386. (doi:10.1016/j.dam.2017.10.007).
18. Tsitsulin, A., Mottin, D., Karras, P., Bronstein, A., Müller, E. 2018 NetLSD: Hearing the shape of a graph. *Proceedings of the 24th ACM SIGKDD International Conference on Knowledge Discovery & Data Mining*. 2347–2356. (doi:10.1145/3219819.3219991).
19. Wills, P., Meyer, F. 2020 Metrics for graph comparison: A practitioner's guide. *PLoS ONE*. **15**.2, 1–54. (doi:10.1371/journal.pone.0228728).
20. Jurman, G., Visintainer, R., Furlanello, C. 2011 An introduction to spectral distances in networks. *Neural Nets WIRN10: Proceedings of the 20th Italian Workshop on Neural Nets*. 227–234. (doi:10.3233/978-1-60750-692-8-227).
21. Ipsen, M., Mikhailov, A. 2002 Evolutionary reconstruction of networks. *Physical Review E*. **66**.4, 046109. (doi:10.1103/PhysRevE.66.046109).
22. Jurman, G., Visintainer, R., Filosi, M., Riccadonna, S., Furlanello, C. 2015 The HIM glocal metric and kernel for network comparison and classification. *Proceedings of the 2015 IEEE International Conference on Data Science and Advanced Analytics, DSAA*. 1–10. (doi:10.1109/DSAA.2015.7344816).
23. Torres, L., Suárez-Serrato, P., Eliassi-Rad, T. 2019 Non-backtracking cycles: Length spectrum theory and graph mining applications. *Applied Network Science*. **4**.1, 41. (doi:10.1007/s41109-019-0147-y).
24. Mellor, A., Grusovin, A. 2019 Graph comparison via the nonbacktracking spectrum. *Physical Review E*. **99**.5, 052309. (doi:10.1103/PhysRevE.99.052309).
25. Schieber, T., Carpi, L., Díaz-Guilera, A., Pardalos, P., Masoller, C., Ravetti, M. 2017 Quantification of network structural dissimilarities. *Nature Communications*. **13928**.8, 1–10. (doi:10.1038/ncomms13928).
26. Bonacich, P. 1987 Power and centrality: A family of measures. *American Journal of Sociology*. **92**.5, 1170–1182. (doi:10.1086/228631).
27. Koutra, D. Vogelstein, J. T., Faloutsos., C. 2016 DeltaCon: Principled Massive-Graph Similarity Function with Attribution *ACM Transactions Knowledge Discovery from Data*. **10**.3, 1–43. (doi:10.1145/2824443).
28. Berlingerio, M., Koutra, D., Eliassi-Rad, T., Faloutsos, C. 2012 NetSimile: A scalable approach to size-independent network similarity. arXiv:1209.2684. (https://arxiv.org/abs/1209.2684).
29. Henderson, K., Gallagher, B., Li, L., Akoglu, L., Eliassi-Rad, T., Tong, H., Faloutsos, C. 2011 It's who you know: Graph mining using recursive structural features *Proceedings of the 17th ACM SIGKDD International Conference on Knowledge Discovery and Data Mining*. 663–671. (doi:10.1145/2020408.2020512).
